# Radiosensitivity index emerges as a potential biomarker for combined radiotherapy and immunotherapy

**DOI:** 10.1038/s41525-021-00200-0

**Published:** 2021-06-02

**Authors:** Yang-Hong Dai, Ying-Fu Wang, Po-Chien Shen, Cheng-Hsiang Lo, Jen-Fu Yang, Chun-Shu Lin, Hsing-Lung Chao, Wen-Yen Huang

**Affiliations:** 1 Department of Radiation Oncology, Tri-Service General Hospital, National Defense Medical Center, Taipei, Taiwan; 2 Department of Radiation Oncology, Wan Fang Hospital, Taipei Medical University, Taipei, Taiwan; 3 Instititue of Clinical Medicine, National Yang Ming Chiao Tung University, Taipei, Taiwan

**Keywords:** Tumour biomarkers, Cancer genomics

## Abstract

In the era of immunotherapy, there lacks of a reliable genomic predictor to identify optimal patient populations in combined radiotherapy and immunotherapy (CRI). The purpose of this study is to investigate whether genomic scores defining radiosensitivity are associated with immune response. Genomic data from Merged Microarray-Acquired dataset (MMD) were established and the Cancer Genome Atlas (TCGA) were obtained. Based on rank-based regression model including 10 genes, radiosensitivity index (RSI) was calculated. A total of 12832 primary tumours across 11 major cancer types were analysed for the association with DNA repair, cellular stemness, macrophage polarisation, and immune subtypes. Additional 585 metastatic tissues were extracted from MET500. RSI was stratified into RSI-Low and RSI-High by a cutpoint of 0.46. Proteomic differential analysis was used to identify significant proteins according to RSI categories. Gene Set Variance Analysis (GSVA) was applied to measure the genomic pathway activity (18 genes for T-cell inflamed activity). Kaplan-Meier analysis was performed for survival analysis. RSI was significantly associated with homologous DNA repair, cancer stemness and immune-related molecular features. Lower RSI was associated with higher fraction of M1 macrophage. Differential proteomic analysis identified significantly higher TAP2 expression in RSI-Low colorectal tumours. In the TCGA cohort, dominant interferon-γ (IFN-γ) response was characterised by low RSI and predicted better response to programmed cell death 1 (PD-1) blockade. In conclusion, in addition to radiation response, our study identified RSI to be associated with various immune-related features and predicted response to PD-1 blockade, thus, highlighting its potential as a candidate biomarker for CRI.

## Introduction

Radiotherapy is one of the most effective treatments for uncomplicated locoregional tumours, and around half of all the cancer patients receive this treatment modality during their course of disease management^1^. With the aid of systems biology, a rank-based radiosensitivity index (RSI) derived from 10 genes (*AR*, *c-JUN* [*JUN*], *STAT1*, *PKC* [*PRKCB*], *Rel A* [*RELA*], *cABL* [*ABL1*], *SUMO1*, *CDK1*, *HDAC1*, and *IRF1*), has been generated to predict the survival fraction at 2 Gy (SF2) across 48 cancer cell lines^2^. The prognostic value of RSI has been validated using several independent data sets, such as those available for the breast and pancreatic cancer, and glioblastoma^3–5^. Moreover, with the inclusion of a linear-quadratic model, the radiation dose could be further adjusted and personalised^6^.

Immunotherapy has revolutionized cancer management since the approval of immune checkpoint inhibitors (ICI) for treatment of metastatic melanoma^7^. Several combinatorial approaches are being investigated to maximize the antitumor immune response, such as radiotherapy combined with immunotherapy (CRI), which possess the potential of inducing durable and synergic effects together. Moreover, over 114 clinical trials involving combined treatment with ICIs and radiotherapy are currently underway^8^. RSI associates important biological networks regulating signalling in response to radiation^9^. Here, we studied the robust association of RSI with intratumoral immune landscape. Moreover, we also identified its correlation with immunotherapy, especially the response to PD-1 blockade. By using whole transcriptomic and matched proteomic data from 12832 primary and 585 metastatic tumours, we aimed to improve decision of the combinatorial therapy.

## Results

### Quality control of RSI across platforms

The output radar plot from sigQC showed that the 10 RSI genes as a signature were characterised by concordant statistical metrics between the Cancer Genome Atlas (TCGA) and Merged Microarray-Acquired dataset (MMD) (Supplementary Fig. 1a). The rank-transformed values for each RSI gene also showed similar distributions (Supplementary Fig. 1b). Taken together, these results suggest that RSI is applicable across these two platforms.

### RSI is altered in tumours

A total of 14502 tumour and normal solid tissues were analysed (Supplementary Fig. 2a). Overall, both tumour and normal tissues demonstrated wide distribution of RSI, with more normal tissues displaying low radiosensitivity. This corresponded to a significantly higher mean RSI in normal tissues in both datasets (0.293 [median = 0.289] vs. 0.340 [median = 0.321] in MMD; 0.537 [median = 0.536] vs. 0.629 [median = 0.633] in TCGA, *p* < 0.001, Supplementary Fig. 2b). Moreover, in different cancer types, we found that the RSI profiles also altered to varied degrees, with significant differential patterns observed in cancer originating in the breast, colon, kidney, and liver (Supplementary Fig. 2c). We also found the differential patterns of RSI gene expression between tumour and normal tissues were specific for these four cancer types (Supplementary Fig. 3a). Further, the RSI is different across cancer types (Supplementary Fig. 3b, ANOVA *p* < 0.001) and the patterns were similar between the TCGA and MMD, even though most TCGA samples were derived from resected early-stage tumours. Similar to clinical observations, pancreatic cancer, kidney cancer, and melanoma showed higher median RSI, whereas liver cancer showed low median RSI. These findings suggest RSI is altered in tumours and could serve as a biomarker in cancer.

### RSI and molecular features associated with immune activation

**Fig. 1:**
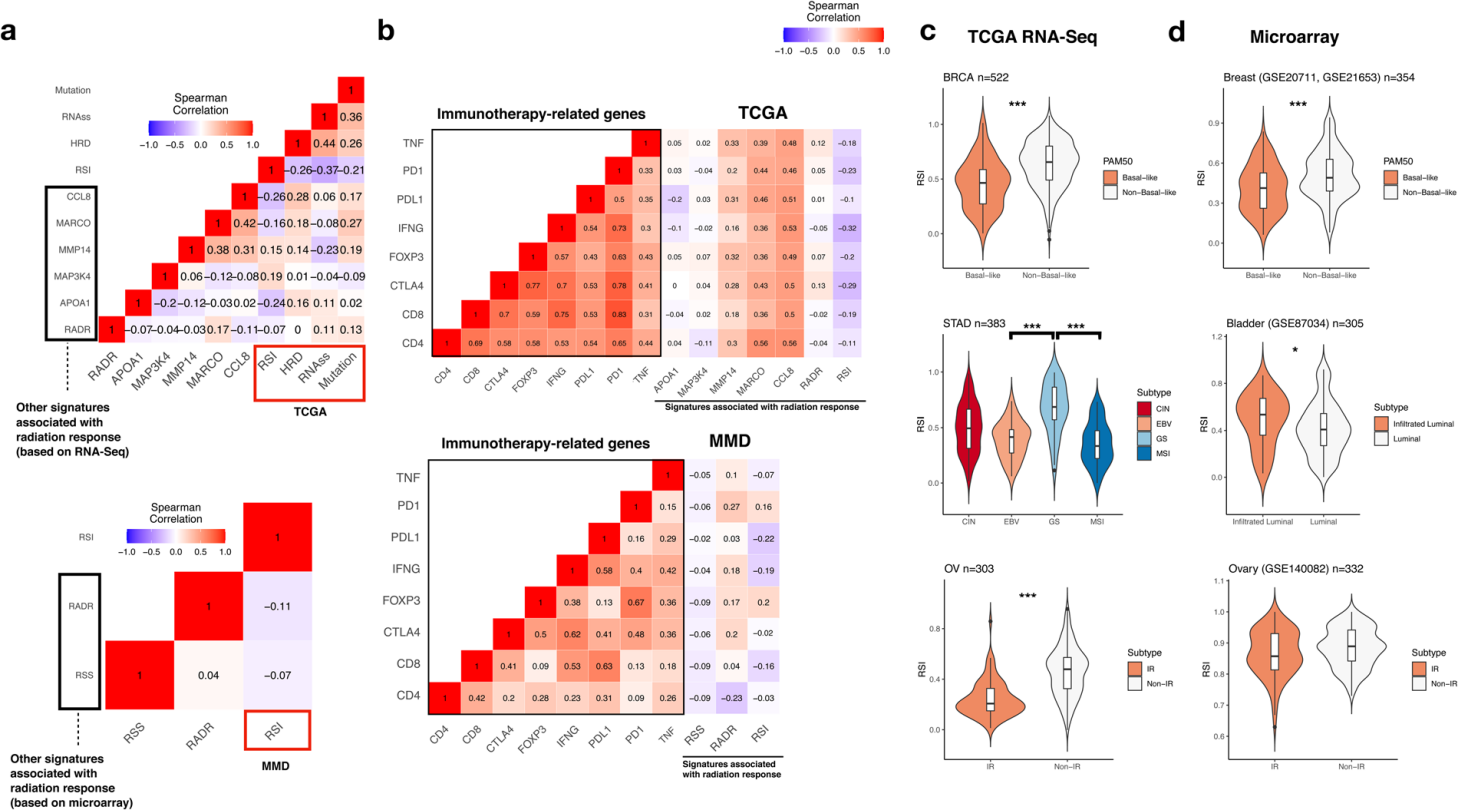
Association of RSI and other gene signatures with immune-related phenotypes and molecular features. **a** Matrix heatmap of Spearman’s correlation among RSI, RADR, RSS, *APOA1*, *MAP3K4*, *MARCO*, *CCL8*, HRD, RNAss, and TMB in TCGA (upper panel) and MMD (lower panel). Colour bar indicates Spearman’s rho. **b** Matrix heatmap of Spearman correlation with immunotherapy-related genes (*TNF*, *PD1*, *PDL1*, *IFNG*, *FOXP3*, *CTLA4*, *CD8*, and *CD4*) in TCGA (upper panel) and MMD (lower panel). Colour bar indicates Spearman’s rho. **c** Association of RSI with molecular subtypes in cancer of the BLCA, BRCA, OV, and STAD in TCGA. **d** Association of RSI with molecular subtypes in cancer of the bladder, breast, ovary, and stomach in MMD. The black horizontal lines represent the median. CIN: chromosomal instability; EBV: Epstein–Barr virus (EBV)-associated; GS: genomically stable; MSI: microsatellite instability; IR: immunoreactive. Mann–Whitney U-test ***p  < 0.001, **p < 0.01, *p < 0.05.

Studies have shown that defective DNA repair, cancer stemness, and mutational burden are associated with radiation as well as antitumor immune response^10,11^. To investigate the relationship between RSI and immune response, we used Homologous recombination deficiency (HRD) and RNA stemness score (RNAss), and total mutation burden (TMB) from TCGA and associated them with RSI and other signatures related to radiation response. Signatures derived from RNA-Seq or microarray were compared, as shown in Fig. 1a, most signatures showed low correlation with RSI in TCGA and MMD datasets except for *CCL8* (Spearman’s rho [rs] = -0.26, *p* = 1.12e-8). The negative correlation of *CCL8* with RSI was partly supported by the negative association of RSI with a 12-chemokine signature which included *CCL8*^9^. In addition, we found that RSI was characterised by a generally stronger negative correlation with HRD and RNAss than other signatures (Spearman’s rs = -0.26, *p* = 2.07e-17; -0.37, *p* = 2.18e-14), although *CCL8* had a higher positive correlation with HRD (Spearman’s rs = 0.28, *p* = 1.96e-12). Furthermore, our analysis also identified RSI to be negatively correlated with TMB (Spearman’s rs = -0.21, *p* = 4.52e-6) across most of the epithelial cancer types (Supplementary Fig. 4). The most negative correlation was observed in three gastrointestinal cancers (colon [Spearman’s rs = -0.2697, *p* = 1.12e-12], pancreas [Spearman’s rs = -0.3512, *p* = 1.45e-10], and stomach [Spearman’s rs = -0.3372, *p* = 2.84e-16]).

We then examined the expression of several immunotherapy-related factors (*CD4*, *CD8A*, *CTLA4*, *FOXP3*, *IFNG*, *PD1*, *PDL1*, and *TNF*). Overall, we found that the expression of *MMP14*, *MARCO*, and *CCL8* signatures was positively correlated with the expression of immunotherapy-related genes. RSI displayed less negative correlations in TCGA, with the strongest negative correlation with *IFNG* (Spearman’s rs = -0.32, *p* = 1.02e-11, Fig. 1b). This negative correlation was also observed in MMD (Spearman’s rs = -0.19, *p* = 3.24e-8). We further identified that the expression of *IFNG* was negatively correlated with RSI in all cancer sites except for prostate cancer in MMD (Supplementary Fig. 5). Of note, *APOA1*, *MAP3K4*, radioresistance score (RADR), and radiosensitivity signature (RSS) correlated poorly with those genes, suggesting that these signatures might have less roles in the crossroads of radiation and immune response.

Using a pre-defined RSI cut-off of 0.46 for tumour stratification, the RSI-Low tumours were found to have significantly higher HRD and RNAss scores than RSI-High tumours (Supplementary Fig. 6). Furthermore, RSI was also significantly associated with different molecular subtypes in cancer (Fig. 1c, d), where tumours with low RSI showed significantly higher proportions of the luminal (bladder), basal-like (breast), immunoreactive (ovary), Epstein–Barr virus (EBV)-associated, and microsatellite instability (MSI) molecular subtypes (stomach) (all *p* < 0.05). Intriguingly, the RSI-Low tumours displayed both molecular features of MSI and higher TMB in stomach cancer, which were shown to be subgroups with favourable outcomes after immunotherapy^12^. With higher HRD scores, RSI-Low tumours could be characterised by higher genome instability and subsequently higher mutational burden. Taken together, RSI-Low tumours may represent a special subpopulation and therapeutic target for immunotherapy.

### RSI and M1 macrophage polarisation

**Fig. 2:**
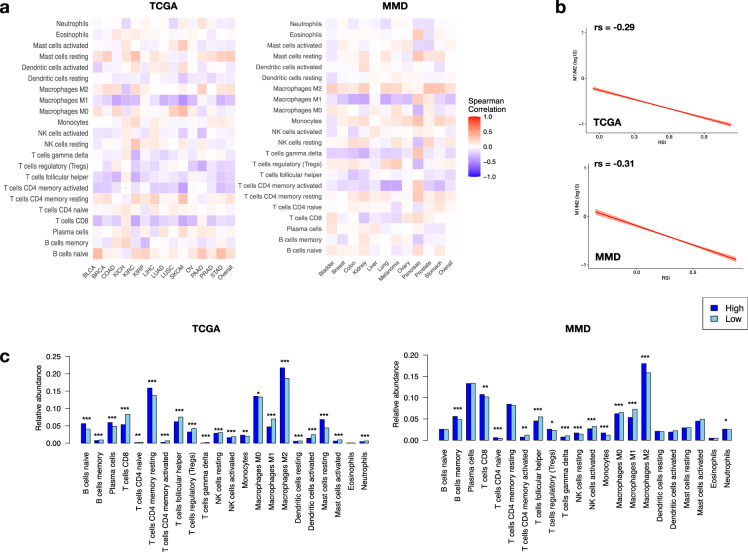
RSI and immune cell infiltrates in tumours. **a** Heatmaps showing Spearman’s correlation between RSI and the relative fraction of 22 immune cell types obtained from CIBERSORT. **b** Correlations between RSI and M1/M2 ratio. Relative portion of M1 and M2 macrophages obtained from (M1/M2) was log2 (M1/M2) transformed for plotting. **c** Distribution of relative abundance of the 22 immune cell subtypes between RSI-High and RSI-Low tumours in TCGA (upper panel) and MMD (lower panel). M1: Macrophage M1; M2: macrophage M2. Mann–Whitney U-test ***p < 0.001, **p < 0.01, *p < 0.05.

Across the 22 immune cell subtypes, we found the fraction of M1 macrophages to be most negatively correlated with RSI (Spearman’s rs = -0.261, *p* = 1.07e-10 in MMD, and -0.248, *p* = 2.16e-18 in TCGA; Fig. 2a). This correlation improved upon examining the relative proportion of M1 and M2 macrophages (M1/2, Spearman’s rs = -0.29, *p* = 1.16e-10 in TCGA, and -0.31, *p* = 2.06e-9 in MMD, Fig. 2b). Additionally, as interferon-γ (IFN-γ) has been shown to induce M1 polarisation (M1P)^13,14^, a strong positive correlation was also observed between intratumoral *IFNG* expression and log2(M1/M2) (Spearman’s rs = 0.532, *p* = 1.69e-19 in TCGA; Supplementary Fig. 7). Additionally, RSI-Low tumours harboured significantly higher portion of follicular T helper cells, T cell gamma delta cells, activated NK cells, and M1 macrophages than RSI-High tumours (Fig. 2c).

The differential expression analysis of 5561 proteins in 95 colorectal adenocarcinoma (COAD) samples indicated high levels of TAP2 (log Fold Change [FC] = -1.33), PARP14 (logFC = -1.25), and GBP4 (logFC = -1.09) in the RSI-Low tumours (Supplementary Fig. 8a and Supplementary Table 1). Concordant with the proteomic analysis, expression of *TAP2*, *PARP14*, *GBP4*, *CKB* and *TRIP6* was significantly high in RSI-Low COAD samples (Supplementary Fig. 8b). TAP1 was included in these differential genes since it forms a heterodimer with TAP2, facilitating antigen presentation to the major histocompatibility complex I (MHCI). Higher expression of these genes was observed in higher M1/M2, especially in cancer of the bladder, breast, colon, melanoma, and stomach (Supplementary Fig. 9). Higher expression of *TAP2* has been reported to associate with improved response to anti-PD-1 treatment^15^. Taken together, RSI-Low tumours were characterised by enhanced antigen presentation machinery and higher M1 proportion, which could lead to pro-inflammatory status and better response to PD-1 blockade.

### RSI and immune landscape in cancers

**Fig. 3:**
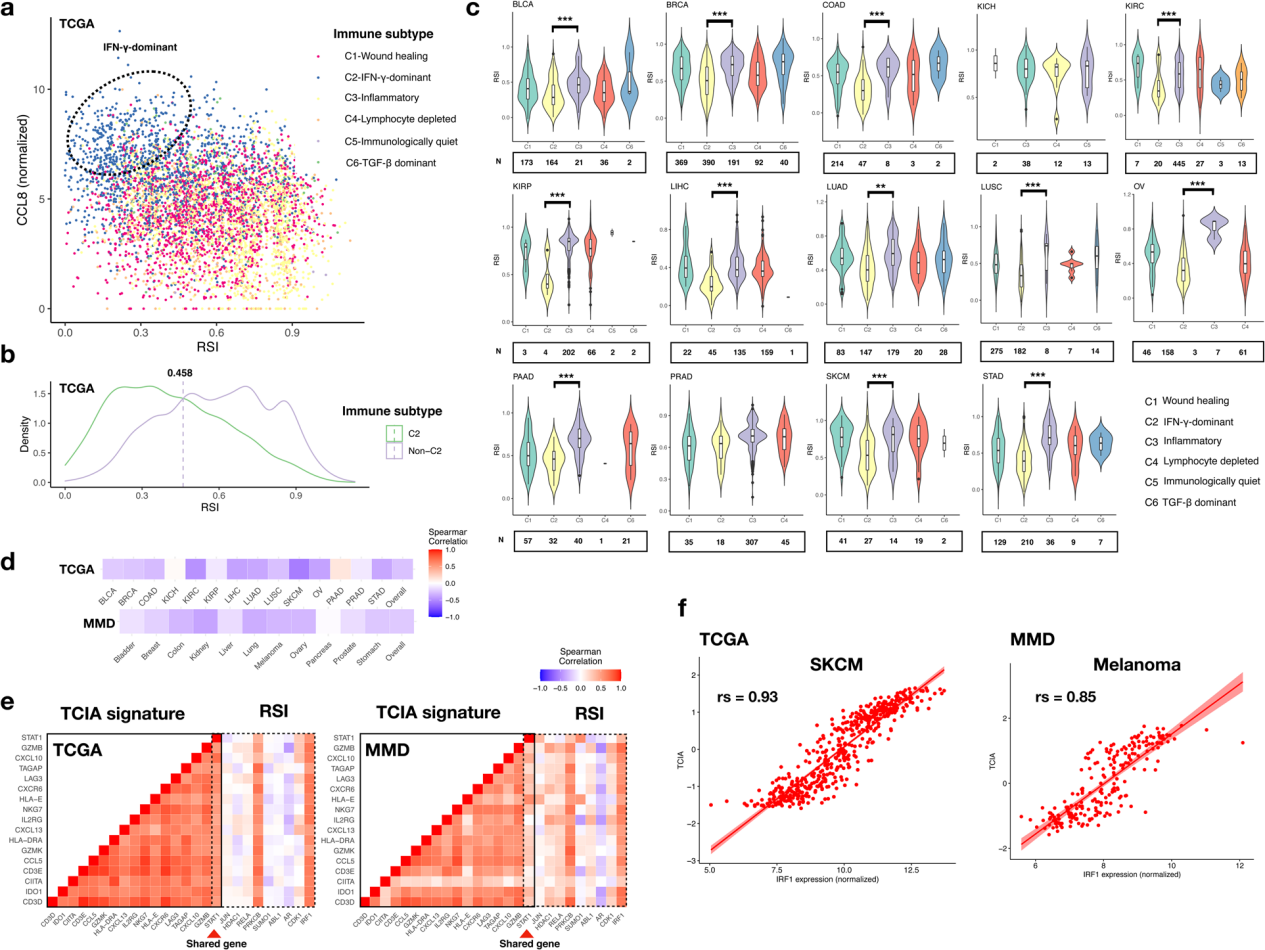
RSI and immune landscape. **a** Scatter plot showing distribution of six immune categories along axes of RSI and *CCL8*. C1, C2, C3, C4, C5, and C6 denote wound healing, IFN-γ-dominant, inflammatory, lymphocyte-depleted, immunologically quiet and TGF-β-dominant pathways, respectively. Dotted circle indicates clustering of the C2 subtype. **b** Density plot showing distribution of RSI in C2 and non-C2 categories. Dotted line indicates the optimal cut-off value. **c** Distribution of RSI in TCGA cohorts separated by immune subtypes. The black horizontal lines represent the median. **d** Spearman’s correlation between RSI and TCIA across the 11 cancer types in TCGA (upper panel) and MMD (lower panel). Colour bar indicates Spearman’s rho. **e** Matrix heatmap of Spearman’s correlation between the 18 TCIA genes and 10 RSI genes in TCGA (left panel) and MMD (right panel). Red arrowhead indicates the overlapped gene between TCIA and RSI. Colour bar indicates Spearman’s rho. **f** Correlations between *IRF1* expression and TCIA score.

To confirm the role of RSI as a candidate signature associated with immunotherapy, we investigated its distribution across different immune categories. We observed that the related RSI and *CCL8* signatures could differentiate C2 (IFN-γ dominant response) from other immune subtypes (Fig. 3a). The density plot also demonstrated a deviated trend of C2 tumours toward lower RSI, with an optimal cut-off value of 0.458, which is very close to the value (0.46) defined previously for RSI stratification (Fig. 3b)^2^.

**Fig. 4:**
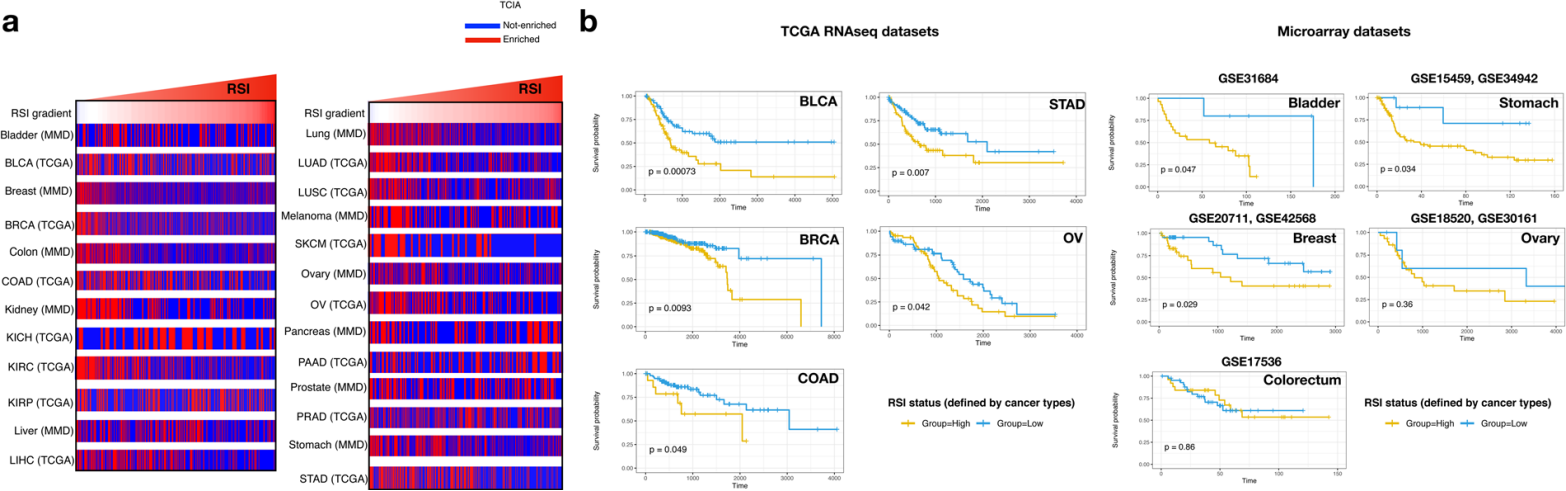
Enrichment of TCIA and survival analysis. **a** Heatmap depicting enrichment status of the TCIA defined by the mean z-transformed GSVA scores > 0.32 in the 11 cancer types. Columns have been ordered by increasing the RSI. **b** KM plots for the OS in patients with cancer of the bladder, breast, colon, ovary, and stomach with enriched TCIA. The RSI-High and RSI-Low tumours have been stratified by the optimal cut-off value for OS in each cancer type.

To identify which RSI genes had a higher impact on this difference, we calculated the feature importance for C2 and non-C2 classifications. Among the 10 RSI genes, the mean feature importance was 177.8 (Supplementary Fig. 10a). *STAT1* was identified to have the highest feature importance (mean decrease in Gini [MDG] = 283.5), followed by *CDK1* (MDG = 236.8), *JUN* (MDG = 228.5), *IRF1* (MDG = 215.4), and *ABL1* (MDG = 180.7). The expression of *JUN*, *CDK1*, *IRF1*, and *STAT1* was significantly higher in the RSI-Low tumours (Supplementary Fig. 10b). A classifier based on *JUN*, *CDK1*, *IRF1*, *STAT1* and *ABL1* showed a high classification accuracy of 82% across all malignancies (95% confidence interval [CI] = 0.798–0.841), with an area under the receiver operating characteristic (ROC) curve of 0.8449 (Supplementary Fig. 10c). In the TCGA dataset, C1 (wound healing) and C2 were the main immune subtypes in most cancer sites, except for renal and prostate cancers, which harboured more C3 (inflammatory) subtypes (Fig. 3c). Instead of KICH and PRAD, tumours in the C2 subgroup were generally associated with significantly lower RSI than those in the C3 subgroup (Fig. 4a). These findings suggest that RSI-Low tumours are mainly dominated by IFN-γ-related responses.

### T cell-inflamed activity (TCIA) is enriched in tumours with low RSI

About 41% tumours in the 11 TCGA cohorts were found to be potentially sensitive to pembrolizumab according to our defined threshold (0.35, Supplementary Fig. 11). We found an overall negative correlation between RSI and TCIA (Spearman’s rs = -0.26, *p* = 2.38e-15 in TCGA; -0.23, *p* = 6.87e-13 in MMD, Fig. 3d). The TCIA signature consisted of 18 genes that intersected RSI genes with *STAT1* (Fig. 3e). The 18 genes showed high intra-correlation in both TCGA and MMD. Moreover, two RSI genes, *PRKCB* and *IRF1*, showed a high positive correlation with the TCIA signature. The highest correlation was found between *IRF1* expression and TCIA in melanoma (Spearman’s rs = 0.93, *p* = 2.12e-15 in TCGA; 0.85, *p* = 4.43e-15 in MMD, Fig. 3f and Supplementary Table 2). Divided by 0.35 to determine enrichment, TCIA was found to be enriched in most RSI-Low tumours (Fig. 4a). The most pronounced enrichment was observed in colon, kidney, lung, and stomach cancers (Table 1).

**Table 1:** Distribution of RSI between TCIA-enrichment and non-TCIA-enrichment.

TCGA	Low (*N*)	High (*N*)	*P*	MMD	Low (*N*)	High (*N*)	*P*
BLCA			<0.001	Bladder			0.264
E	133	45		E	63	14	
NE	140	108		NE	100	35	
BRCA				Breast			
E	176	292	<0.001	E	770	124	0.87
NE	115	625		NE	1208	200	
COAD				Colon			
E	69	76	<0.001	E	479	111	<0.001
NE	47	132		NE	643	281	
KICH				Kidney			
E	2	38	0.303	E	93	39	<0.001
NE	7	44		NE	55	136	
KIRC				Liver			
E	132	126	<0.001	E	149	8	0.3978
NE	54	293		NE	225	19	
KIRP				Lung			
E	12	115	0.029	E	379	25	<0.001
NE	16	189		NE	501	92	
LIHC				Melanoma			
E	154	100	<0.001	E	64	27	0.504
NE	145	22		NE	80	43	
LUAD				Ovary			
E	138	93	<0.001	E	213	45	0.068
NE	94	249		NE	309	96	
LUSC				Pancreas			
E	142	89	<0.001	E	24	48	0.582
NE	149	172		NE	30	76	
SKCM				Prostate			
E	16	15	<0.001	E	104	0	0.075
NE	4	69		NE	127	6	
OV				Stomach			
E	97	32	<0.001	E	263	41	0.009
NE	81	93		NE	341	92	
PAAD							
E	21	53	0.586				
NE	36	72					
PRAD							
E	22	203	1				
NE	32	292					
STAD							
E	127	62	<0.001				
NE	91	170					

*RSI* radiosensitivity index, *TCIA* T-cell inflamed activity, *E* Enrichment, *NE* non-enrichment.

### RSI is predictive of survival in several cancer types

Univariate analysis in the entire TCGA cohort showed that tumours with high RSI and TCIA associated with favourable survival outcomes (Hazard ratio [HR] = 0.29, 95% CI = 0.23–0.36, *p* < 0.001 for RSI; HR = 0.86, 95% CI = 0.82–0.91, *p* < 0.001 for TCIA; Supplementary Fig. 12). However, using the optimal cut-off for overall survival (OS) in tumours enriched in TCIA (Supplementary Table 3), cancer of the bladder, breast, and colon with low RSI displayed favourable survival outcomes (Fig. 4b), whereas unfavourable outcomes were observed in cancer of the kidney and prostate (Supplementary Fig. 13).

### Metastatic tumours are characterised by low RSI with varied TCIA

**Fig. 5:**
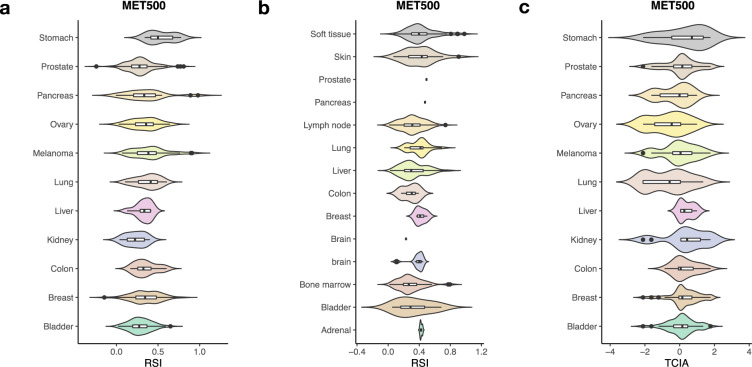
RSI in the metastatic tissues. **a** RSI in metastatic tissues in the MET500 cohort. **b** RSI of tumours in various metastatic tissues. **c** TCIA scores in the 11 cancer types. The black horizontal lines represent the median.

In the MET500 database, the median RSI was found to be less than 0.5, in 10 cancer types (Fig. 5a and Supplementary Table 4). Bone marrow was the most sensitive metastatic site, with higher RSI observed for sites in organs, such as the breast, brain, adrenal gland, and lung (Fig. 5b). For metastases originating from the bladder, breast, and prostate (all N > 50), the median TCIA was above 0 (Fig. 5c). Similar to the primary tumours, even with low RSI, enrichment of TCIA was only achieved in 32.9 % (Bladder, 26/79), 43.4% (Breast, 69/159), and 34.2% (Prostate, 53/155), respectively.

## Discussion

In the large-scale analysis of RSI across 11 major cancer types, we identified that RSI was related to various immune-relevant genomic and molecular features, and low RSI were associated with dominant IFN-γ signalling response and predicted therapeutic efficacy of PD-1 blockade.

Tumours with defective DNA repair response (DDR) tend to have accumulation of genomic errors, which may trigger increased presentation of tumour-specific neoantigens^16^. In the context of MHCI, the neoantigens are specifically recognized by T cells, enhancing the antitumor immune response^10^. In urothelial cancers, mutations in DDR pathways are associated with better efficacy of PD-1/PD-L1 blockade^17^. Here we demonstrated lower RSI correlated with higher HRD score and higher TMB, suggesting the presence of deficient DNA repair mechanism and potential of responding to immune-based therapies^18^. HRD score was associated with genes involved in homologous repair, including *BRCA1*, *BRCA2*, *RAD51B*, and *RAD51C*, and alteration of these genes are linked to radiosensitivity^19,20^. Additionally, lower RSI also correlated with higher RNAss, which indicated higher degrees of stemness and tumour de-differentiation^21^. Malta et al. reported higher RNAss is associated with increased PD-L1 protein expression in BLCA, BRCA, COAD, LUAD, KICH, KIRC, KIRP, OV, PAAD, and STAD cohorts in TCGA, suggesting potential effectiveness to ICI for these tumours.

Immune responses are activated in few molecular subtypes, such as the basal-like, immunoreactive, and EBV-associated subtype in cancer of the breast, ovary, and stomach, respectively^22–24^. These immune-associated subtypes show inter-similarity, and are partly characterised by increased CD8 + T cell infiltrates and up-regulated IFN-γ signalling signatures^25–27^. Additionally, compared to other molecular features, these subtypes associate with a significantly low RSI. Furthermore, the expression of IFNG, which translates to the actionable IFN-γ response, was negatively correlated with RSI, supporting the up-regulation of IFN-γ signalling activity in these immune-associated subtypes^28^. Tobin et al. utilized 12 chemokine genes to define the intratumoral immune activation and identified that low RSI significantly associates with high immune activation (using an RSI cut-point of 0.3745)^9^. Interestingly, *CCL8* was listed as one of the chemokine genes, supporting its negative correlation with RSI in the present study. Furthermore, as RSI genes such as *STAT1* and *IRF1* are downstream of IFN-γ-mediated signalling, RSI has a better correlation with various immune-related molecular features and phenotypes than other genes and gene signatures related to radiation response^29^.

Tumours with C2 subtype were characterised by the highest M1P, highest CD8 + T cells, and high proliferation rate^30^. Concordant with this phenotype, here, the RSI-Low tumours showed high percentage of M1 macrophages. Despite an opposite trend observed for the CD8 + T cells in the MMD, RSI-Low tumours also harboured more follicular T helper cells, T cell gamma delta cells, activated NK cells. Of note, these cell types are all capable of IFN-γ secretion, suggesting a dominant role of IFN-γ in RSI-Low tumours^31–33^. Studies suggest that IFN-γ could transform macrophages to a proinflammatory phenotype and induce M1P^34,35^. When IFN-γ binds receptor on macrophages, it induces STAT1 homodimerisation, which triggers IFN-γ-dependent signalling^36,37^. The canonical IRF/STAT signalling is central in modulating the macrophage polarisation. Additionally, IFN-γ induces synthesis of IRF1^38^, which plays an important role in inflammation, immunity, cell proliferation, and apoptosis^38,39^. Interestingly, *STAT1* and *IRF1* have been listed in the RSI genes, thus associating radiosensitivity with response to IFN-γ. Further, in the IFN-γ dominant microenvironment, the antigen processing machinery, including MHCI, TAP1, and TAP2 may upregulate^40,41^. The TAP1 and TAP2 facilitate MHCI antigen presentation^42^, which subsequently activate the CD8 + T lymphocytes. The IFN-γ released by activated CD8 + T lymphocytes primes the macrophages toward the M1 phenotype^43^. Here, the intratumoral expression of TAP1 and TAP2 showed higher clustering with increasing M1 population in several cancer types. Therefore, the mechanism regulating this phenomenon may be contributed by common action of local IFN-γ response.

An increased expression of PD-L1 on the surface of tumour cells or immune cells has been associated with an improved response to the PD-1 checkpoint blockade^44^. However, single measurement of PD-L1 would limit the understanding of the interaction between cancer and immune cells^45^. Therefore, genomics analyses have been proposed to elucidate the complex tumour microenvironment (TME)^46–48^. M Ayers et al. utilized patient cohorts from three KEYNOTE trials and confirmed the robustness of a pan-tumour T cell-inflamed gene signature in predicting the response to PD-1 blockade^49^. The TCIA, defined by the gene signature, is characterised by IFN-γ signalling, cytotoxic effector molecules, antigen presentation, and T cell active cytokines. This “hot” immune-inflamed TME associates with high CD8 + lymphocytes, myeloid cells, monocytic cells, high IFN levels, and stimulation of chemokines, including CXCL9, CLCL10, and CXCL11, which predict benefit from the PD-1 blockade^50^. Here, we observed enrichment of TCIA in tumours with low RSI, which could be correlated to favourable OS in several cancer types. However, IFN-γ plays a dual role in cancer progression^51^, and upon prolonged activation of IFN-γ in the TME, the tumour cells may develop resistance, thus activating tumorigenic pathways^41^. Moreover, upregulation of PD-L1/2 by IFN-γ in cancer, stromal, and myeloid cells would lead to immune evasion^52^. Furthermore, the C2 subtype was found to associate with a less favourable outcome in the TCGA cohort. In addition to the edited immune response, the absence of survival benefit in several tumours with low RSI, and the corresponding high portion of C2 subtypes could be explained by their aggressiveness and the associated high proliferation^30^. Furthermore, tumours with re-modelled immune response show loss of genes responsible for antigen presentation, making them less immunogenic^30^. Therefore, the C2 category is of prognostic value in immunotherapy. The classifier based on five RSI genes, *STAT1*, *CDK1*, *JUN*, and *IRF1*, and *ABL1* sufficiently predicted the presence of the C2 subtype. Taken together, the identification of tumours with the C2 subtype could better characterise the immune status while assessing possible radiation responders.

To enhance the efficacy of PD-1 blockade, selecting tumours with upregulated PD-L1/2 expression is of prognostic significance, especially in tumours with increased exposure to IFN-γ^41,53^. Additionally, inhibition of the PD-1/PD-L1 axis would prevent exhaustion of CD8 + T cells, and enhance response to immunotherapy^54^. CRI seems promising, as radiation may help induce immunogenic cell death, elicit innate immune system, and promote antigen presentation to infiltrating CD8 + T cells^41,55^. Here, the RSI-Low tumours were found to associate with active IFN-γ signalling, M1P, upregulated antigen presentation machinery, response to PD-1 blockade, and response to radiotherapy. Therefore, CRI appears to be the most effective therapy for RSI-Low tumours.

There were some technical limitations in our study. First, we could not obtain the serial RSI change for tumours. Therefore, we could not elucidate the change of radiosensitivity overtime during the radiotherapy. Second, the immune cell infiltration was only the estimation, not the real TME. The TME is complicated might not purely reflect the real fractions of immune cells from RNA sequencing. Third, the metastatic tissues from MET500 dataset were not sufficient for statistical analysis and comparison with the primary tumours, limiting the use of RSI in this situation.

Despite these limitations, with the help of large sample cancer genomics dataset, our study identified strong association between RSI and IFN-γ response and immunotherapy, although future work would be required to elucidate the detailed mechanism and role of RSI in CRI.

## Methods

### Data source

MMD used in our study have been previously described^56^. The datasets cover 11 major cancer types (bladder, breast, colon, liver, lung, kidney, melanoma, ovary, pancreas, prostate, and stomach), and comprise of 95 independent Gene Expression Omnibus (GEO) studies (http://www.ncbi.nlm.nih.gov/geo) and a total of 8386 samples, either tumours or relevant normal tissues. Raw data for the 11 cancer types were independently pre-process using author-defined methods or RMA-normalization using the R library *affy* package^57^. All the raw data were based on the GPL 570 microarray platform (Affymetrix Human Genome U133 Plus 2.0 Array) and were merged and adjusted by the Combat method using the R library *inSilicoMergine* package^58^. Probes annotated with specific genes were collapsed to the maximum expression values, which were adopted for subsequent analyses.

Normalized RNA-Seq data based on Illumina HiSeq platform were extracted for the 11 cancer types and 14 cohorts from the Cancer Genome Atlas (TCGA; abbreviation: BLCA, BRCA, COAD, LIHC, LUAD, LUSC, KICH, KIRC, KIRP, OV, PAAD, PRAD, SKCM, STAD) using the bioinformatics tool Xena browser (https://xenabrowser.net/). Here, rectal adenocarcinoma was included in COAD, which was then used for comparison with colorectal tissues in MMD. Raw RNA-Seq data were quantified using the root square error method (RSEM), and log_2_ transformed (RSEM + 1). The associated clinical parameters, such as survival and molecular subtypes were obtained for comparative purpose. Finally, 6116 tumour (primary tumour) and normal (solid tissue normal) tissues were retrieved.

Raw RNA-Seq data in MET500 for the 11 epithelial carcinoma were downloaded from the database of Genotypes and Phenotypes, subsequently processed using RSEM^59,60^, and then normalized using fragments per kilobase of transcript per million mapped reads (FPKM) and log_2_ transformed (FPKM + 0.001). A total of 585 metastatic tumours from various cancer types and body locations (soft tissue, skin, prostate, pancreas, lymph node, lung, liver, colon, breast, brain, bone marrow, bladder, and adrenal gland) were analysed.

### Signature quality control

As RSI was derived from microarray datasets, its use in RNA-Seq platforms has never been elucidated. Prior to proceeding further, we first evaluated the quality of RSI application on TCGA and MMD using *sigQC*^61^*. sigQC* is an R package for gene signature quality control, which encompasses a number of statistical metrics describing the ability of a gene signature to represent a dataset of interest, such as variability of signature genes and co-correlation of signature genes.

### Calculation of RSI signature

RSI was calculated using a rank-based linear regression model. 10 RSI genes were indexed from the entire genomics data in the MMD and TCGA, and the expression values were reordered in the respective platforms. Gene with the highest expression value was ranked 10. The RSI was constructed using the following equation:

1 $$\begin{array}{ll}{\mathrm{RSI}} =\!\!\!\! & - \,0.0098009 \ast AR + 0.0128283 \ast JUN\\ &+ \,0.0254552 \ast STAT1 - 0.0017589 \ast PRKCB - 0.0038171 \ast RELA\\ &+\, 0.1070213 \ast ABL1 - 0.0002509 \ast SUMO1\\& -\, 0.0092431 \ast CDK1 - 0.0204469 \ast HDAC1 - 0.0441683 \ast IRF1\end{array}$$

The RSI value corresponded to the SF2, and therefore a low RSI suggests high radiosensitivity. Furthermore, a previous study used ROC and found a cut-off point of 0.46, which was associated with the best predictive accuracy in pathological response after radiotherapy^2^. Based on this value, tumours were grouped into RSI-High and RSI-Low, which essentially represent two different types of tumours in terms of response to radiotherapy.

### Gene signatures associated with radiation response

To investigate the role of RSI as a promising candidate in linking radiotherapy and immunotherapy, we searched for other gene signatures or genes reported to predict radiation response. A 34-gene RSS was developed and validated by Cui et al. This signature was derived from a microarray platform and was shown to predict the benefit of radiotherapy in breast cancer^62^. RSS was calculated in MMD according to the author’s method. Another 13-gene RADR was developed by Foy et al. and was shown to predict recurrence after radiotherapy in head and neck squamous cell carcinoma^63^. It was calculated using a single-sample gene set enrichment analysis tool and was applied well in both RNA-Seq and microarray platforms. In addition to the radiotherapy-associated scores, we searched for genes relevant to the radiation response. In locally advanced breast cancer, *APOA1*, *MAP3K4*, and *MMP14* were differentially expressed in the responders in the neoadjuvant setting^64^. *MARCO* and *CCL8*, which are associated with immune infiltration and radiation outcome, were also included^65^. For comparative analyses, RSS and RADR were applied in MMD, whereas RADR, as well as *APOA1*, *MAP3K4*, *MMP14*, *MARCO*, and *CCL8* were applied to the TCGA RNA-Seq platform.

### HRD and RNAss

The HRD and RNAss were retrieved from analytic data type in the Xena browser using the TCGA-Pan-Cancer dataset. HRD and RNAss scores were used to represent the degree of HRD and cancer stemness.

### Molecular subtypes, TMB and immunotherapy-related genes

To assess the association of RSI with immune-related molecular subtypes in cancers, seven independent datasets with annotated molecular features were obtained (Bladder: accession number = GSE87034 in GEO; Breast: GSE20711 and GSE21653, and BRCA in TCGA; Ovary: GSE140082, and OV in TCGA; Stomach: STAD in TCGA). In cancer originating in the bladder, breast, and ovary, the RSI was dichotomized based on the molecular subtypes associated with immune response. The four molecular subtypes characterised by TCGA in gastric adenocarcinoma^22^, based on genome stability and infection, were considered for our study to associate with RSI and immune responses. Furthermore, immunotherapy-related factors, including *CD4*, *CD8A*, *CTLA4*, *FOXP3*, *IFNG*, *PD1*, *PDL1*, and *TNF* were examined and correlated with RSI. TMB for tumour samples from the TCGA was retrieved from the Genomic Data Commons Data Portal (http://portal.gdc.cancer.gov/projects/). Here, the TMB was defined as the total number of simple somatic mutations observed in each of the TCGA cancer cohorts. Tumours containing at least one mutation were included.

### CIBERSORT and polarisation of macrophages

Relative fractions of distinct immune cell types found in the TME were estimated using the beta version of the CIBERSORT (http://cibersort.stanford.edu/). Next, 1000 tumours were randomly selected from cancers originating in the breast, colon, and lung as their gene expression data in the MMD exceeded the 500 Mb file quota. Further, the LM22 signature gene file and 500 permutations were selected as our input parameters. The quantile normalization was disabled for runs with the RNA-Seq data. The LM22 signature gene file contains 547 genes that accurately distinguish 22 types of immune cells, including the T cells, B cells, plasma cells, and NK cells with different activation states, and various subsets of the myeloid lineage, facilitating an overview of immune cells infiltrating the tumours. Moreover, for each immune cell subtypes, the Spearman’s correlation was calculated between the relative levels of immune cells and RSI. Next, the polarisation of macrophages was assessed from the data generated in CIBERSORT. The M1P status was determined using a ratio of the M1 and M2 macrophages, which was obtained based on the fraction of their relative amounts, and was log_2_ (M1/M2) transformed and correlated with RSI using the Spearman’s rs test.

### Proteomic and differential analysis

To investigate the impact of RSI on the protein level, we conducted a proteomic analysis using available data from the NCI Clinical Proteomic Tumor Analysis Consortium (https://cptac-data-portal.georgetown.edu/cptac/s/S016). Relative protein abundance data of 5562 genes for matched colorectal cancer cohort of the TCGA was obtained (TCGA_Colon_VU_Proteome_CDAP_Protein_Report.r2). The cohort comprised of 95 samples (64 colon and 31 rectum tumour tissues). Unshared spectral count values were used to represent the relative protein abundance. The R library *edgeR* package was used to assess differential protein expression between the RSI-High and RSI-Low tumours^66^. FC > 1 or FC < − 1 and adjusted *p* < 0.05 were used to determine the significantly differential proteins. Volcano plot was generated using the R library *EnhancedVolcano* package.

### Gene set variation analysis (GSVA)

TCIA, which associates with response to the PD-1 blockade, was described as an immunotherapy-related pathway by our group^49^. GSVA scores of this pathway, inclusive of 18 genes, were computed for each tumour sample in the MMD and TCGA datasets. The R/Bioconductor *GSVA* package was used to calculate these scores, which were then transformed to z-scores^67^. We applied a cut-off of 0.35 for mean z-score, which was previously identified in melanoma^46^, to determine whether the samples in our study were enriched or not for TCIA. A heatmap was used for visualization of the correlation between the TCIA enrichment status and RSI.

### Determining IFN-γ dominant response

To establish a classifier for C2 subtype, feature importance of the RSI genes in determining IFN-γ dominant response was first quantified using the MDG^68^. The MDG applies the Random Forest algorithm (RF) and utilizes the Gini impurity to measure the feature importance. An increase in MDG correlates with a decrease in node impurity, and thus enhanced importance. Genes with MDG above mean feature importance were used for building a classifier. Binary classification of the IFN-γ-dominant response subtype (C2) was based on an optimal cut-point determined by the R library *cutpointr* package. Classifier of the C2 subtype was established using the RF algorithm, and the resultant performance of the model was estimated using the area under the ROC curve with 95% CI. The calculation of MDG and construction of the RF-based classifier were performed using the R library *randomForest* package.

### Survival analysis

Survival data obtained from the TCGA and other independent GEO datasets were analysed using the R library *survival* package. The RSI allowed stratification into RSI-High and RSI-Low groups based on the optimal cut-point using the *surv_cutpoint* function in *survminer* package. Kaplan–Meier (KM) survival curve was derived for OS according to RSI. The HR of RSI and TCIA for OS was calculated and plotted using the hr_plot function in the R library *finalfit* package. The log-rank test was used to identify survival differences between the groups.

### Statistical analysis

All statistical analyses were conducted in R. For comparison of variables under defined conditions, the Mann–Whitney U-test was used to derive the *p*-value. One-way ANOVA was used for multiple comparisons of RSI across cancer types. For both statistical analyses, *p-*value < 0.05 was considered statistically significant. The density plot was used to observe the estimated distribution of RSI between the normal and tumour tissues. For comparisons of different datasets (MMD and TCGA), the *p* values derived for RSI genes for normal vs. tumour tissues were quantified and -log_10_(*p*) transformed, and were then visualized by bubble plot.

### Reporting summary

Further information on research design is available in the Nature Research Reporting Summary linked to this article.

## Supplementary information

Supplementary Information

Reporting Summary

## Data Availability

All data generated or analysed during this study are available from the corresponding author on reasonable request. Data from TCGA could be downloaded from Xena browser (https://xenabrowser.net/). Data for MET500 could be retrieved from database of Genotypes and Phenotypes (accession number: phs000673.v2.p1). Processed cancer type-specific MMDs are available from ArrayExpress under accession numbers (MTAB-6690, E-MTAB-6691, E-MTAB-6692, E-MTAB-6693, E-MTAB-6694, MTAB-6695, E-MTAB-6696, E-MTAB-6697, E-MTAB-6698, E-MTAB-6699, and E-MTAB-6703). Proteomic data for colorectal cancer are available from the NCI Clinical Proteomic Tumor Analysis Consortium (https://cptac-data-portal.georgetown.edu/cptac/s/S016).

## Code availability

The scripts used for the bioinformatics reanalysis are available at https://github.com/YangHongDai/Radiosensitivity_index.
