# Supplementary material for: Radiosensitivity index emerges as a potential biomarker for combined radiotherapy and immunotherapy
Source: NPJ Genom Med. 2021 Jun 2;6:40. doi: 10.1038/s41525-021-00200-0 (PMC8172905; doi:10.1038/s41525-021-00200-0)
Supplement: Supplementary file 1 — Supplementary Information [file 41525_2021_200_MOESM1_ESM.pdf]

Supplementary Figures and Tables

Radiosensitivity index emerges as a potential biomarker for combined radiotherapy and immunotherapy

Yang-Hong Dai, Ying-Fu Wang, Po-Chien Shen, Cheng-Hsiang Lo, Jen-Fu Yang, Chu-Shu Lin, Hsing-Lung Chao and Wen-Yen Huang

Supplementary Figure 1

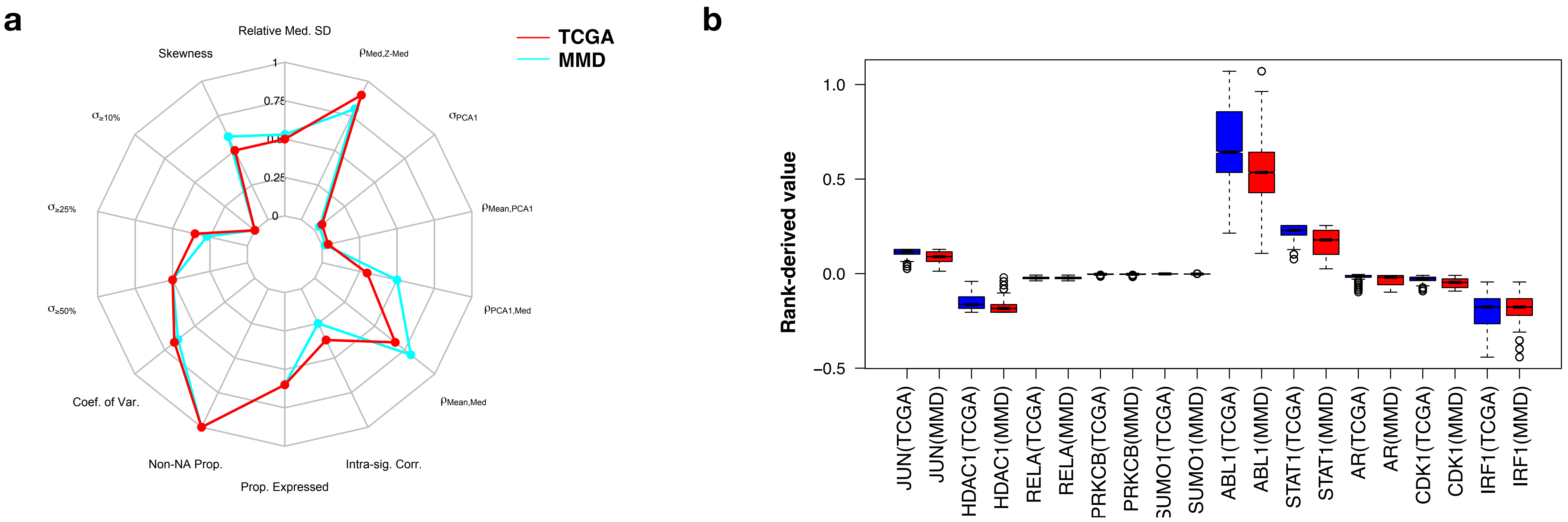

Supplementary Figure 1: Signature quality control in TCGA and MMD.

a Radar plot produced from sigQC. Each ray of the radar plot represents one of the summary metrics for RSI as a gene signature. **b** Distribution of each rank-transformed RSI genes in TCGA and MMD. The box hinges represent the 1st and 3rd quartiles, while the middle black horizontal lines indicate the median.

Supplementary Figure 2

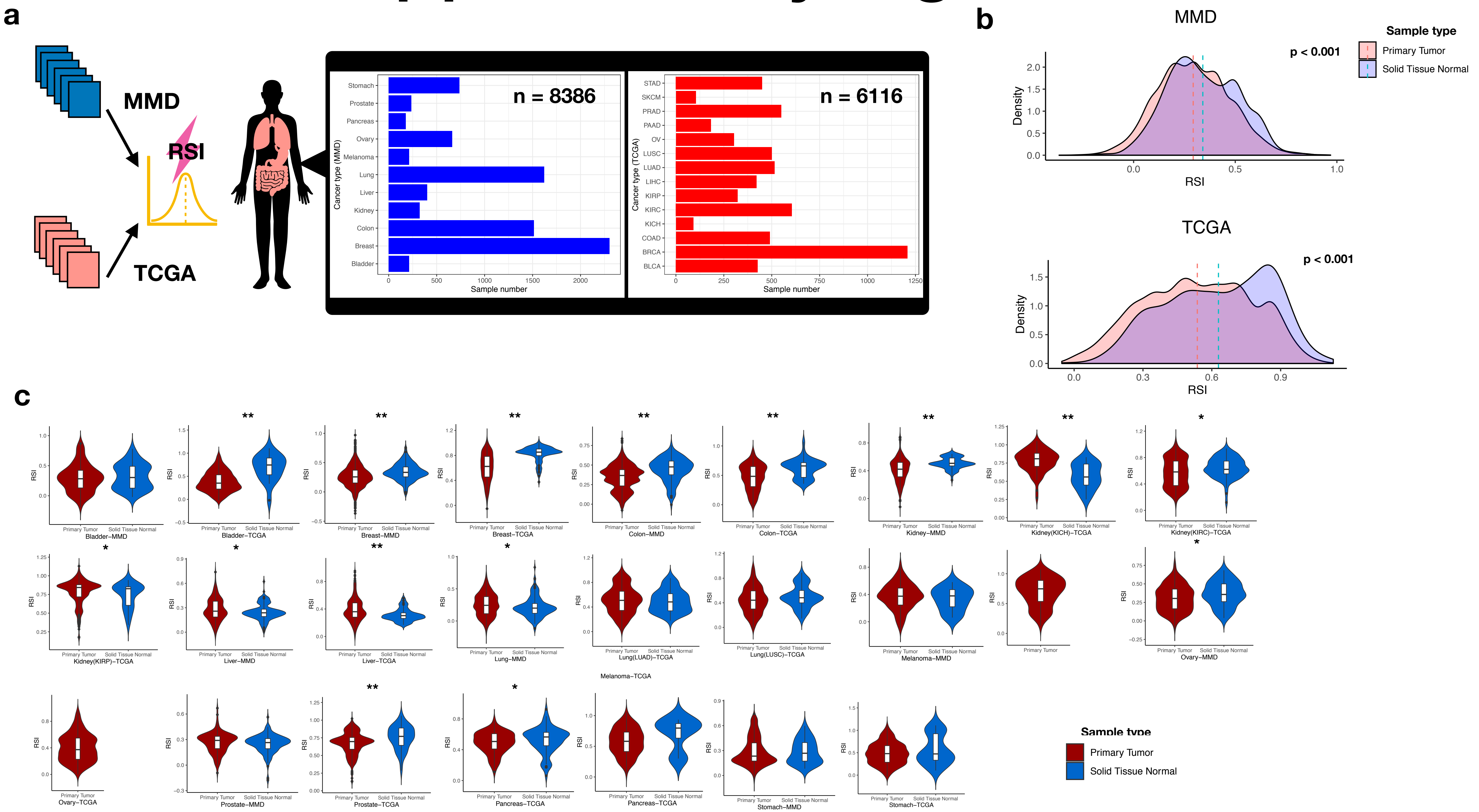

Supplementary Figure 2: Variation of RSI in 11 major cancer types.

a Illustration of datasets used for characterization of RSI. **b** Density plot analyses depicting RSI distribution in tumour (MMD: n = 7286, TCGA: n = 5546) vs. normal tissues (MMD: n = 1100, TCGA: n = 570). Red and green horizontal dotted lines represent the mean RSI values in the primary tumour and normal tissues, respectively. **c** RSI in normal (solid normal tissue) vs. tumour (primary tumour) tissues across 11 cancer types. The black horizontal lines represent the median. Mann–Whitney U-test ***p < 0.001, **p < 0.01, *p < 0.05. As melanoma cohort in TCGA does not contain normal tissues, only the tumour tissues are shown.

Supplementary Figure 3

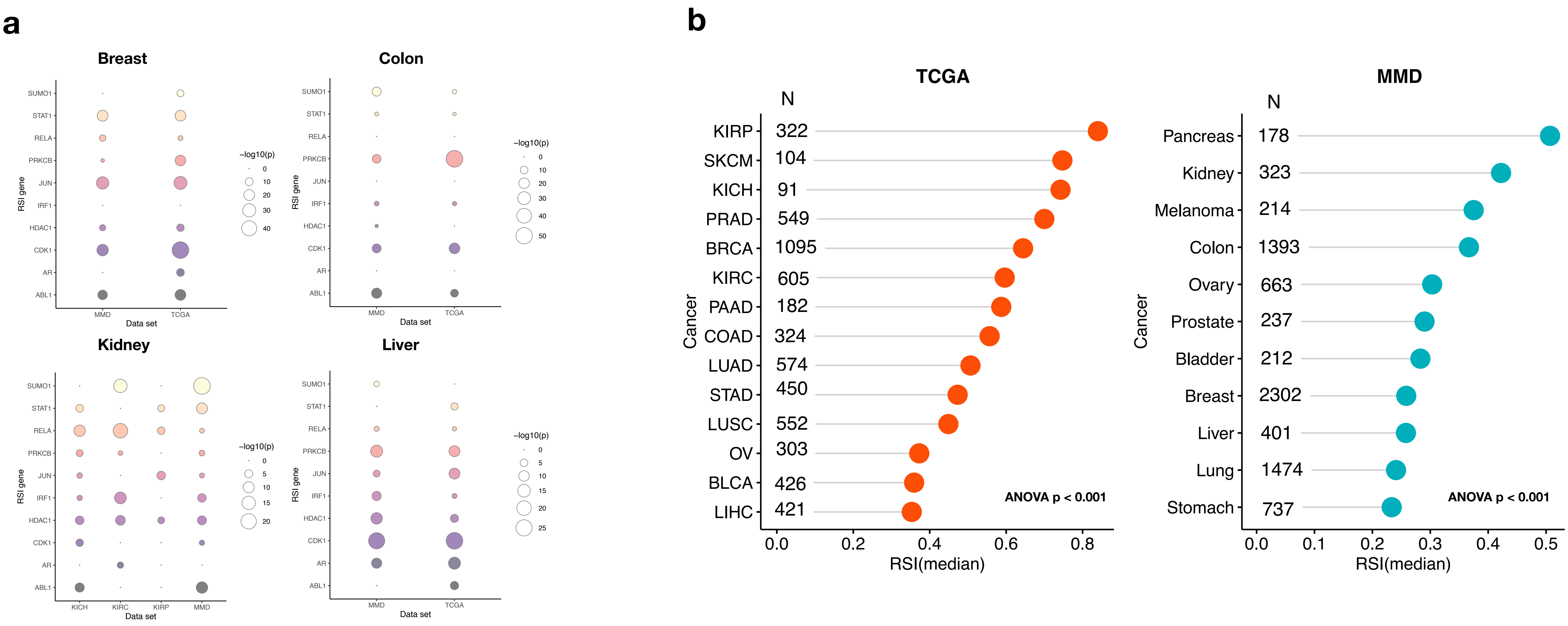

Supplementary Figure 3: Differences of RSI between tumour and normal tissues and across cancer types. a RSI gene differences between normal and tumour tissues. p value derived from the Mann–Whitney U-test is transformed to -log10(p). **b** Ranking of median RSI value across the 11 cancer types in TCGA (left panel) and MMD (right panel).

Supplementary Figure 4

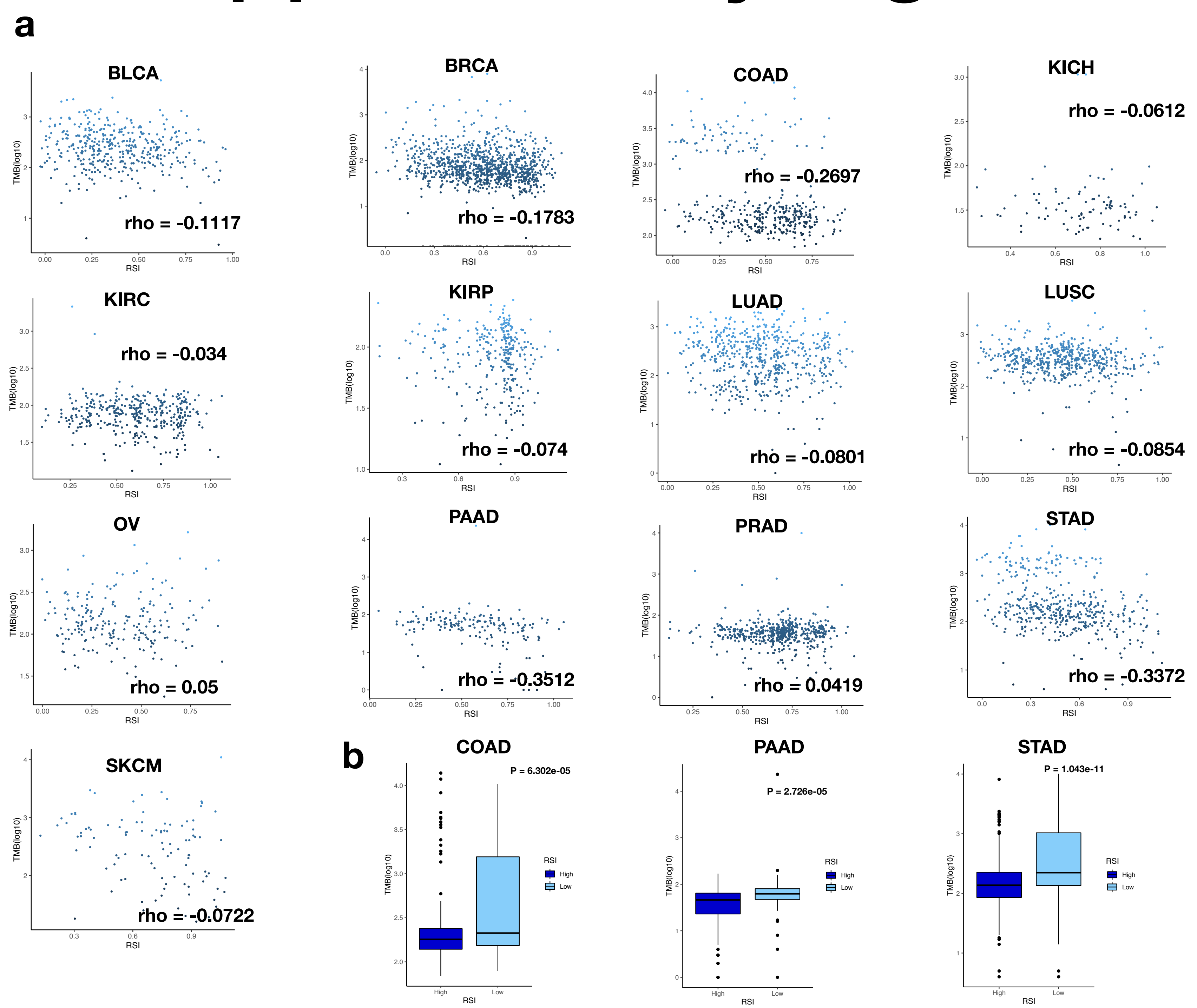

Supplementary Figure 4: Correlation of RSI with TMB.

a Spearman's correlation between RSI and TMB in the TCGA. TMB was log10-transformed for plotting. **b** Distribution of log10(TMB) between RSI-High and RSI-Low tumours in COAD, PAAD and STAD.

Supplementary Figure 5

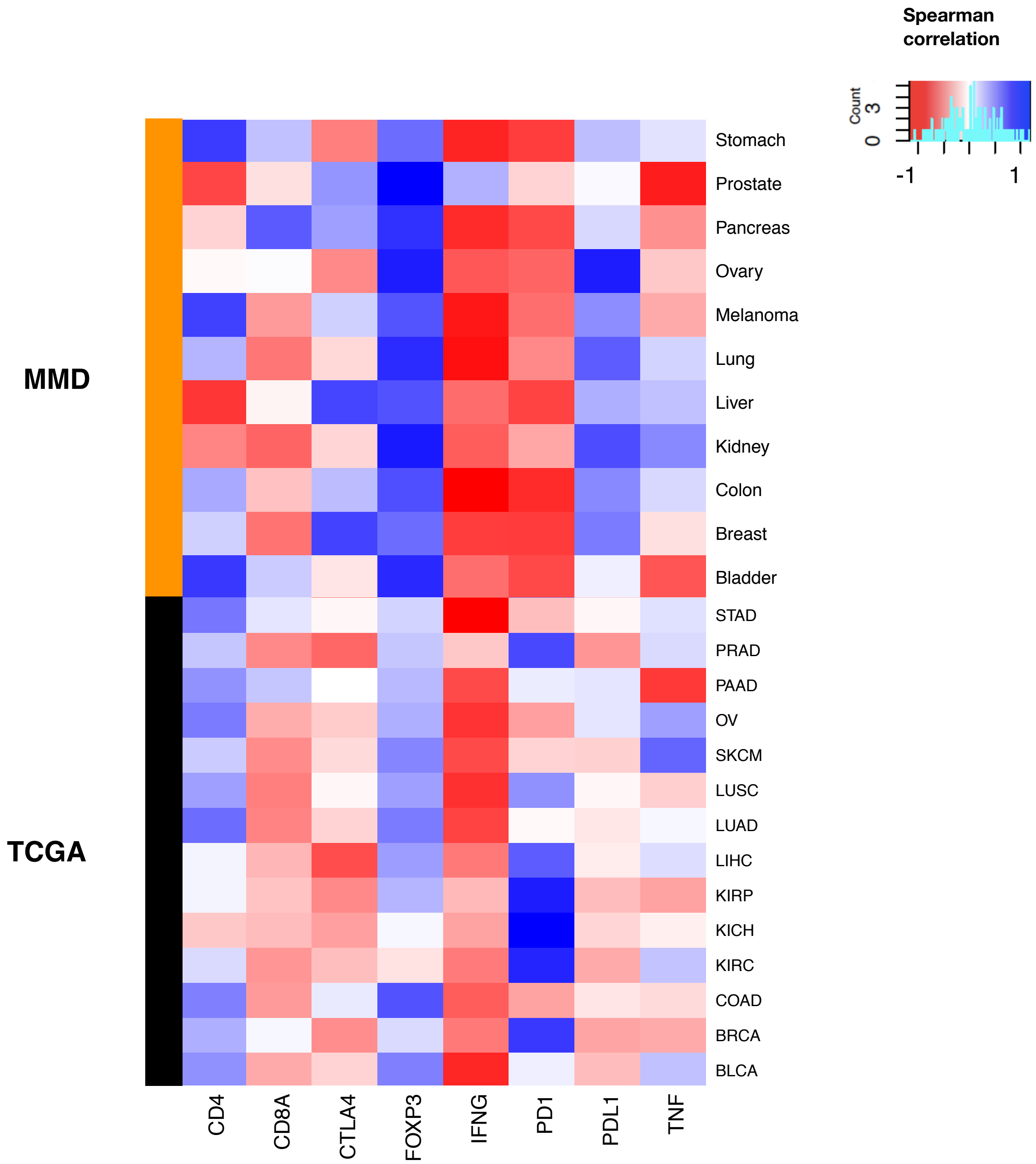

Supplementary Figure 5: Heatmap depicting Spearman's correlation between RSI and expression of genes related to immunotherapy across 11 major cancer types in TCGA and MMD. Colour bar indicates Spearman's rs.

Supplementary Figure 6

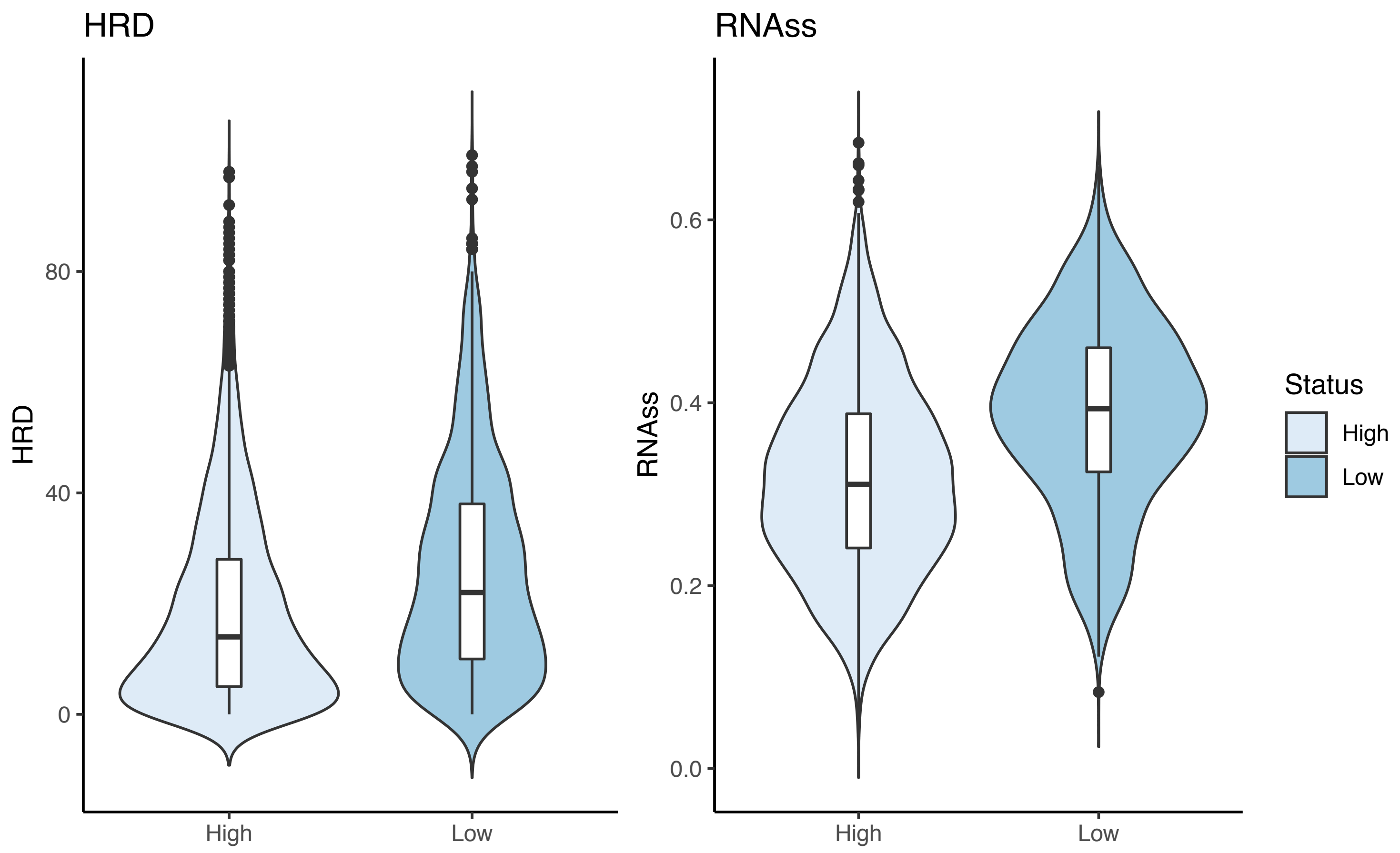

Supplementary Figure 6: Violin plots showing distribution of HRD and RNAss scores between RSI-High and RSI-Low tumours in TCGA. The black horizontal lines represent the median.

Supplementary Figure 7

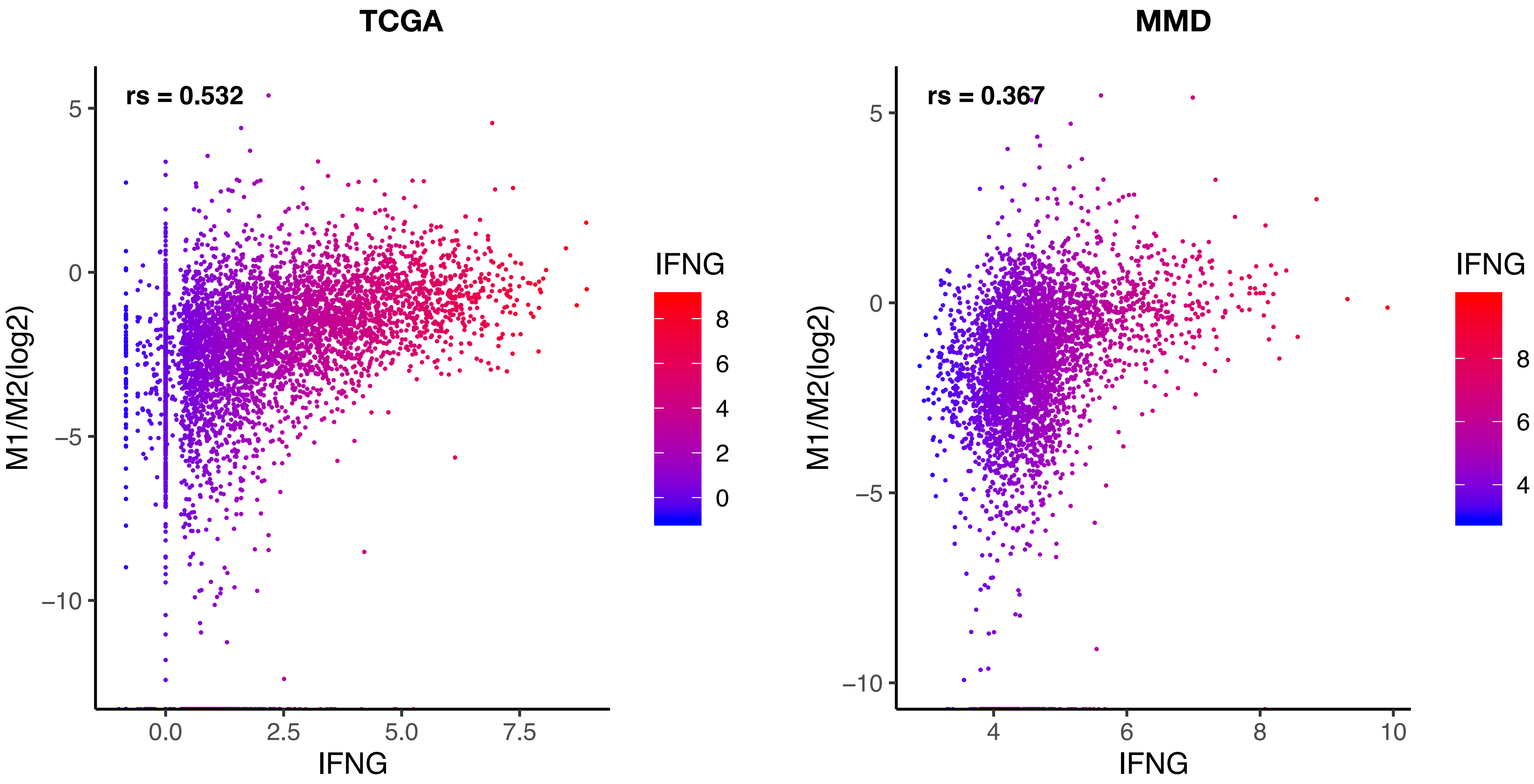

Supplementary Figure 7: Scatter plots showing correlation between *IFNG* expression and M1/M2 (log2) in TCGA (upper panel) and MMD (lower panel). M1: Macrophage M1; M2: macrophage M2.

Supplementary Figure 8

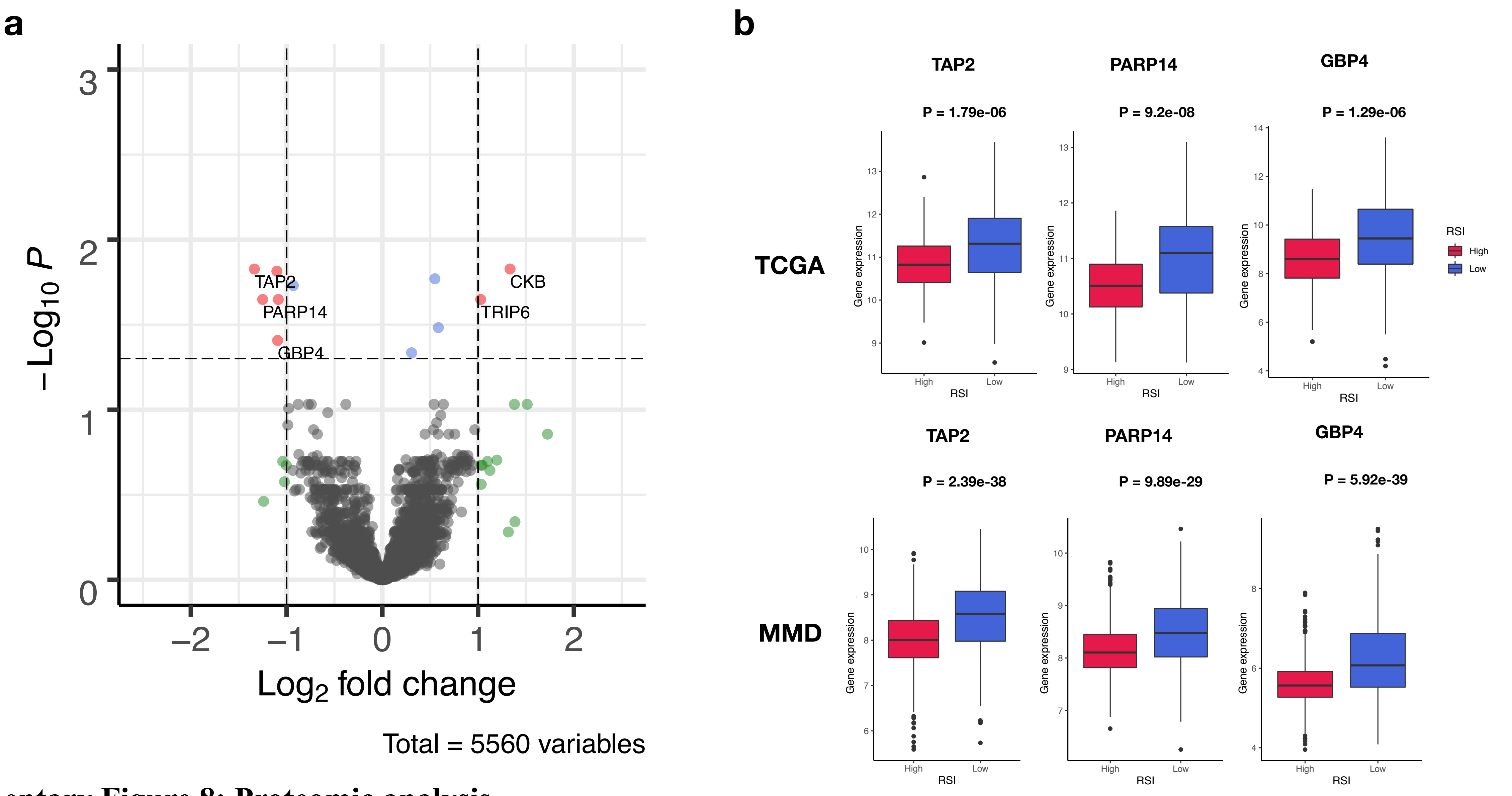

Supplementary Figure 8: Proteomic analysis.

a Volcano plot illustrating differentially expressed proteins in the RSI-High and RSI-Low tumours. Red dots indicate proteins with FC > 1 or < -1 and adjusted *P* < 0.05. Blue dots represent proteins with -1 < FC < 1 and adjusted *P* < 0.05. Green dots represent proteins with FC > 1 or < -1 and adjusted *P* > 0.05. (B) Box plots showing expression of *TAP2*, *PARP14* and *GBP4* between RSI-High and RSI-Low tumours in TCGA (upper panel) and MMD (lower panel). The box hinges represent the 1st and 3rd quartiles, while the middle black horizontal lines indicate the median.

Supplementary Figure 9

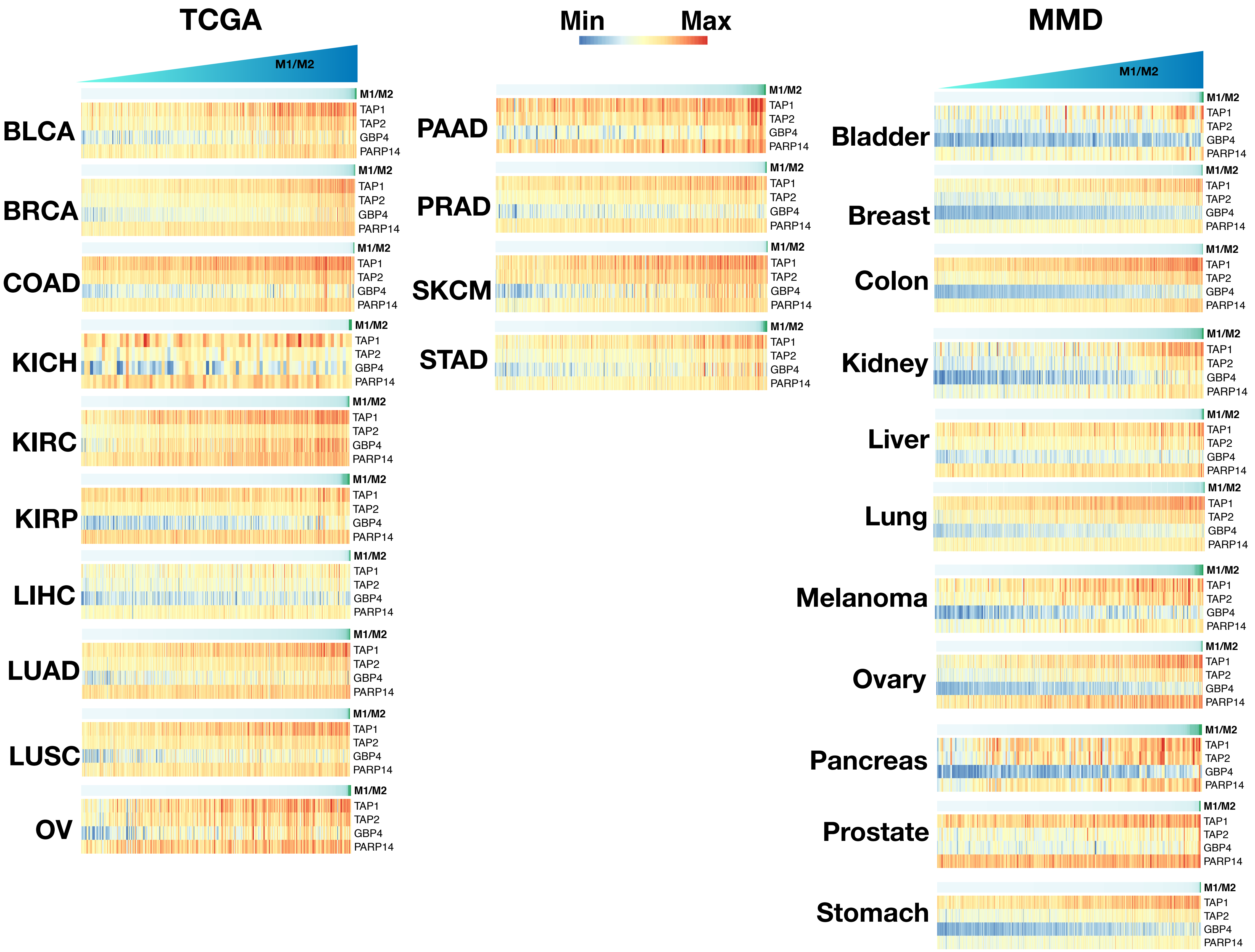

Supplementary Figure 9: Heatmaps showing gene expression level of *TAP1*, *TAP2*, *PARP14*, and *GBP4*. Columns have been ordered based on increasing M1/M2 ratio. M1: Macrophage M1; M2: macrophage M2.

Supplementary Figure 10

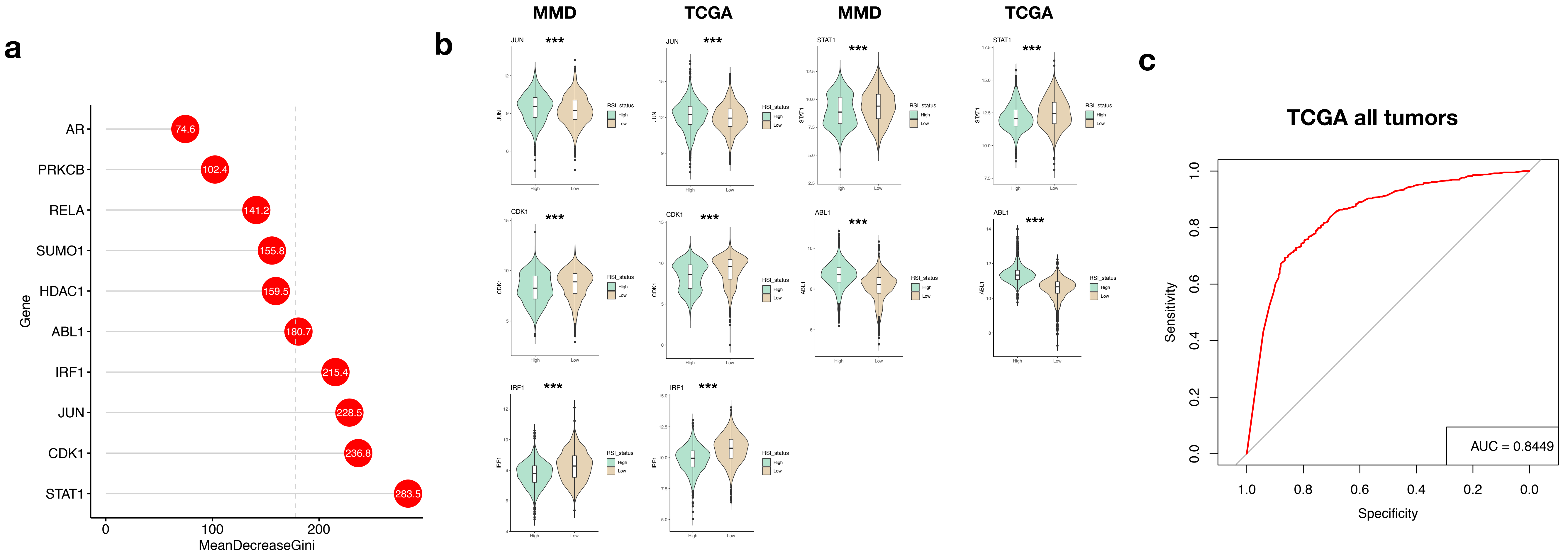

Supplementary Figure 10: Classifier of C2 immune subtype.

a Mean decrease in Gini for the 10 RSI genes in classification of C2 subtype. Gray horizontal dotted line indicates a MDG of 177.8. **b** Expression of *JUN*, *CDK1*, *IRF1*, *STAT1* and *ABL1* between RSI-High and RSI-Low tumours in the TCGA (left panel) and MMD (right panel). **c** Receiver operating characteristic curve for the established model in predicting C2 subtype.

Supplementary Figure 11

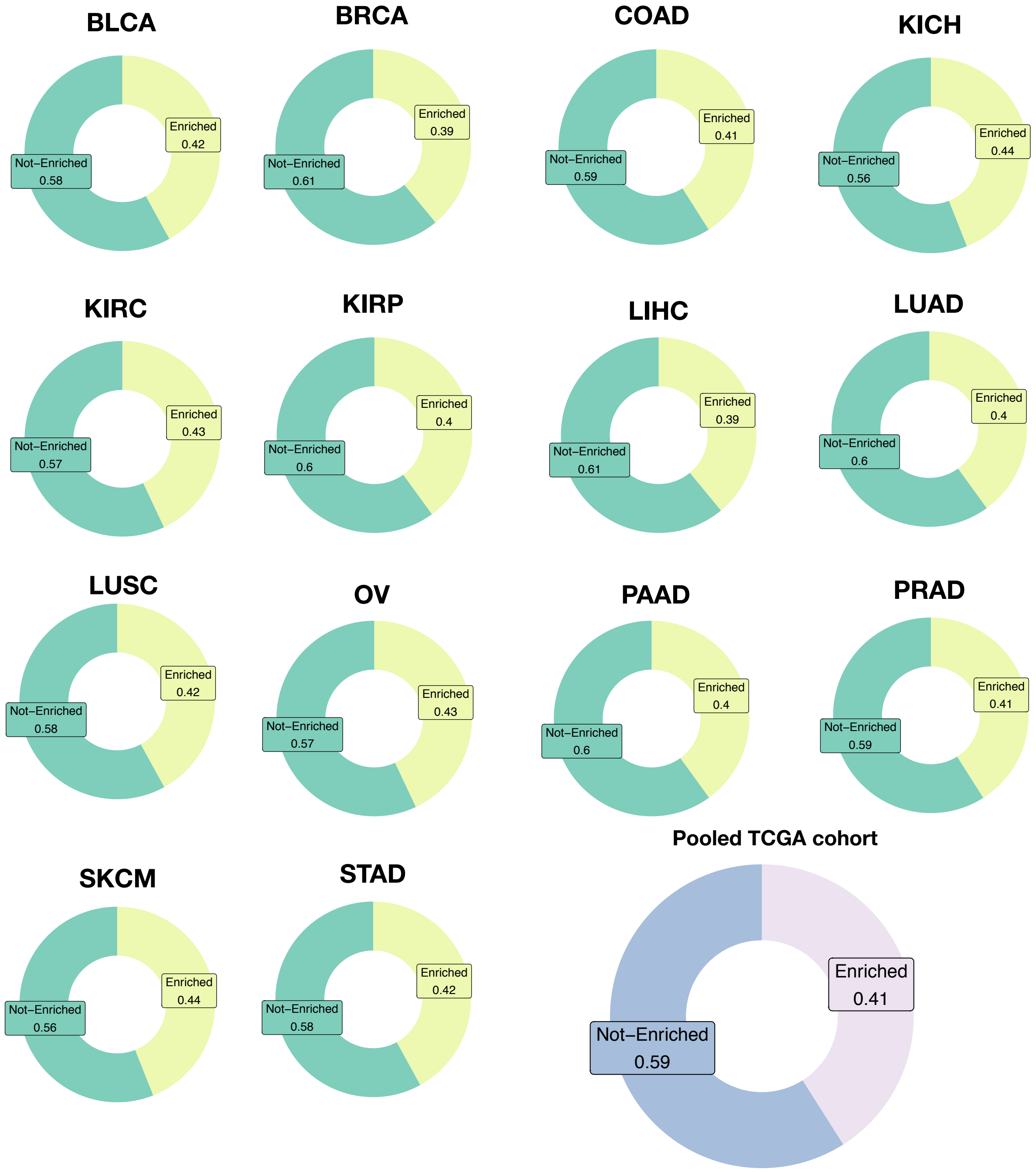

Supplementary Figure 11: Donut plots depicting percentages of TCIA-enriched and not-enriched tumour samples in the 11 TCGA cancer cohorts.

Supplementary Figure 12

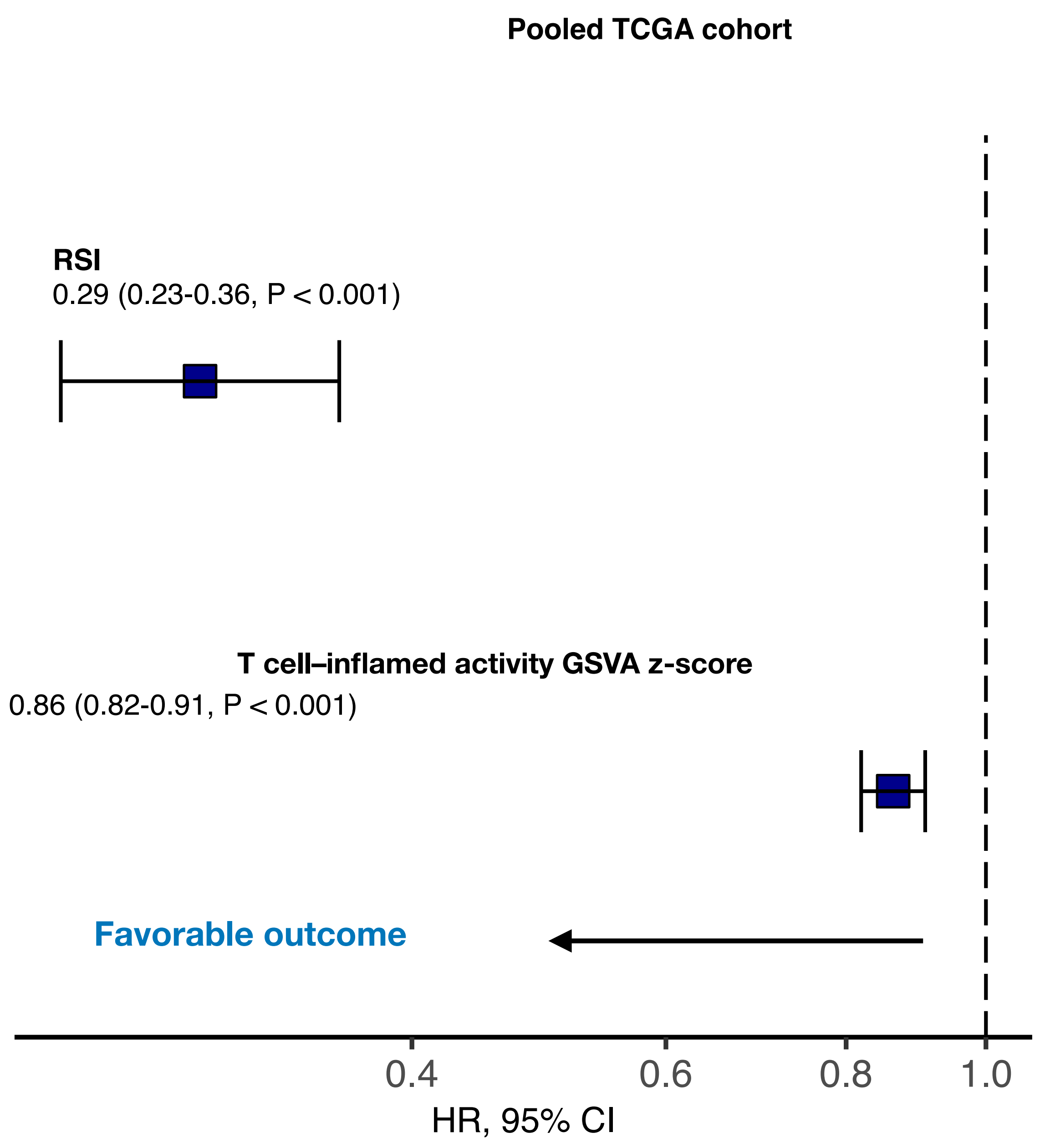

Supplementary Figure 12: Hazard ratio plot indicating favourable overall survival with high RSI and TCIA in the pooled TCGA cohort with the 11 cancer types.

Supplementary Figure 13

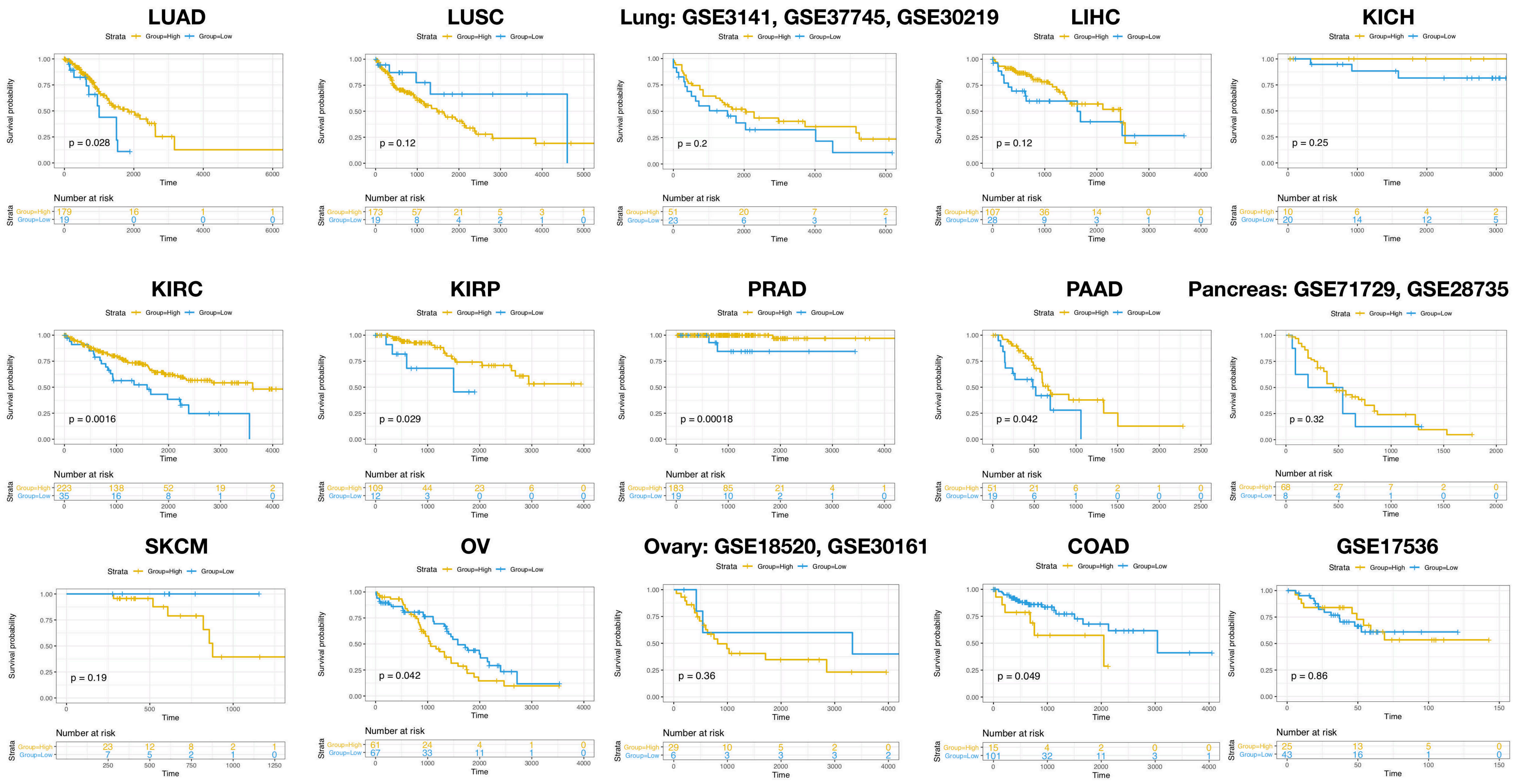

Supplementary Figure 13: KM plots for lung, liver, kidney, prostate, pancreas and melanoma in TCGA and several independent GSE datasets

Supplementary Table 1

Gene	logFC	AveExpr	t	P.Value	adj.P.Val	B
TAP2	-1.33635858039912	7.10180369178457	-5.11273123854195	2.89055437531139E-06	0.0148879408907804	4.1468920630629
PARP14	-1.24968104709633	7.47887540925286	-4.47535117580896	3.05853208850455E-05	0.0224818108451525	2.21254110804622
MUC5B	-1.23948925911655	7.88186030972953	-2.38345382119777	0.0200122762472588	0.345763214669471	-3.37630331791356
GBP1	-1.10098891036227	8.110312201045	-4.83292146198769	8.27350604401098E-06	0.0153335645349003	3.46223052006926
GBP4	-1.09360011426999	5.5409463892827	-4.24043684911711	7.04094578702984E-05	0.0391476585758859	1.31701594031414
RNF213	-1.08685217483235	9.58144205106456	-4.45974864744149	3.23479292736007E-05	0.0224818108451525	2.11348967238193
PARP9	-1.04034227475808	6.61737476219293	-3.23671570492533	0.00188608139222394	0.201202774300534	-1.35269161283532
OAS2	-1.0214865963295	5.99474331809927	-2.69841907169059	0.00882110189611531	0.265250375564611	-2.58502642187831
DDX60	-1.00284567795184	6.59803792963941	-2.99419713225748	0.00386044765501153	0.211937027554233	-1.9301218249963
HMGCS2	1.72493369206382	8.13507237087143	3.5036321728	0.000825699589811233	0.139117870283347	-0.635191248904549
DPEP1	1.51267783629714	6.62112429112653	3.82506076617208	0.00029091648498259	0.0929517040292831	0.135648011947727
FCGBP	1.38495196886674	9.96849827015208	2.09622043278546	0.039868513722781	0.455407305085665	-4.36422096278626
AMACR	1.37938425012813	6.68174738248085	3.8438754480137	0.000273262116239093	0.0929517040292831	0.179160483378131
CKB	1.3336970142633	8.35457843800319	4.94940567399612	5.35537442114403E-06	0.0148879408907804	3.82443185620885
CLCA1	1.31572439269828	8.31327930865293	1.86973594807095	0.0659205369499829	0.523651212473593	-4.32987512594169
CES1	1.19643096492599	7.9362310078049	3.26258325465329	0.00174392060847022	0.197881603736621	-1.27630600890553
FBN2	1.12336671810833	6.85852022804601	2.8553219694421	0.00572841257413255	0.227434234390211	-2.24598057823757
EPB41L1	1.09824612068415	6.43169502363832	3.22313827935965	0.00196498660760428	0.201202774300534	-1.41203185340778
PTK7	1.04292763813684	7.00302726599441	3.03885384417029	0.00339206335657135	0.211937027554233	-1.82660192599739
ANPEP	1.03264175954616	6.74636232542563	2.66910230053988	0.00954580772926303	0.274653401837071	-2.64615703549093
LTBP2	1.03036057310203	7.12019301455174	2.98140415713139	0.00400530792343813	0.211937027554233	-1.95774735288577
TRIP6	1.02714214311488	5.64410483473386	4.48091858415269	2.99792515338385E-05	0.0224818108451525	2.06292403610948

Supplementary Table 1: Differential expression analysis for proteomics in colorectal cohort in TCGA. Genes with absolute logFC > 1 are shown.

Supplementary Table 2

Cancer_type	STAT1	PRKCB	IRF1
BLCA (TCGA)	0.78	0.56	0.8
Bladder (MMD)	0.49	0.56	0.58
BRCA (TCGA)	0.68	0.82	0.75
Breast (MMD)	0.71	0.81	0.76
COAD (TCGA)	0.71	0.7	0.78
Colon (MMD)	0.53	0.68	0.65
KICH (TCGA)	0.15	0.44	0.5
KIRC (TCGA)	0.67	0.7	0.8
KIRP (TCGA)	0.36	0.64	0.64
Kidney (MMD)	0.7	0.79	0.8
LIHC (TCGA)	0.57	0.79	0.62
Liver (MMD)	0.6	0.74	0.56
LUAD (TCGA)	0.7	0.66	0.77
LUSC (TCGA)	0.71	0.72	0.83
Lung (MMD)	0.65	0.69	0.8
SKCM (TCGA)	0.79	0.71	0.93
Melanoma	0.79	0.59	0.85
OV (TCGA)	0.54	0.49	0.56
Ovary (MMD)	0.63	0.52	0.64
PAAD (TCGA)	0.56	0.77	0.56
Pancreas (MMD)	0.66	0.84	0.74
PRAD (TCGA)	0.61	0.5	0.68
Prostate (MMD)	0.34	0.36	0.41
STAD (TCGA)	0.61	0.54	0.74
Stomach (MMD)	0.64	0.67	0.66
Overall (TCGA)	0.6	0.64	0.71
Overall (MMD)	0.6	0.67	0.66

Supplementary Table 2: Correlation of TCIA and three RSI genes across tumours.

Supplementary Table 3

Cancer types	RSI
BLCA (TCGA)	0.29
Bladder (GSE31684)	0.13
BRCA (TCGA)	0.56
Breast (GSE20711, GSE42568)	0.22
LUAD (TCGA)	0.15
LUSC (TCGA)	0.1
Lung (GSE3141, GSE37745, GSE30219)	0.25
LIHC (TCGA)	0.2
OV (TCGA)	0.28
Ovary (GSE18520, GSE30161)	0.1
COAD (TCGA)	0.67
Colorectum (GSE17536)	0.54
KICH (TCGA)	0.81
KIRC (TCGA)	0.26
KIRP (TCGA)	0.42
PAAD (TCGA)	0.26
Pancreas (GSE71729, GSE28735)	0.58
PRAD (TCGA)	0.44
STAD (TCGA)	0.43
Stomach (GSE15459, GSE34942)	0.14
SKCM (TCGA)	0.31

Supplementary Table 3: Optimal cut-off RSI values for OS.

Supplementary Table 4

Cancer types	N	Median RSI
Bladder	79	0.27
Breast	155	0.35
Lung	42	0.41
Liver	10	0.33
Ovary	24	0.35
Colon	20	0.32
Kidney	12	0.22
Pancreas	25	0.36
Prostate	155	0.28
Stomach	15	0.49
Melanoma	44	0.38

Supplementary Table 4: Median RSI value for metastatic tissues.
