# Supplementary material for: Radiosensitivity index emerges as a potential biomarker for combined radiotherapy and immunotherapy
Source: NPJ Genom Med. 2021 Jun 2;6:40. doi: 10.1038/s41525-021-00200-0 (PMC8172905; doi:10.1038/s41525-021-00200-0)
Supplement: Supplementary file 2 — Reporting Summary [file 41525_2021_200_MOESM2_ESM.pdf]

nature research

Corresponding author(s): Wen-Yen Huang

Last updated by author(s): Mar 25, 2021

Reporting Summary

Nature Research wishes to improve the reproducibility of the work that we publish. This form provides structure for consistency and transparency in reporting. For further information on Nature Research policies, see our Editorial Policies and the Editorial Policy Checklist.

Statistics

For all statistical analyses, confirm that the following items are present in the figure legend, table legend, main text, or Methods section.

n/a	Confirmed	
☐	☒	The exact sample size (*n*) for each experimental group/condition, given as a discrete number and unit of measurement
☐	☒	A statement on whether measurements were taken from distinct samples or whether the same sample was measured repeatedly
☐	☒	The statistical test(s) used AND whether they are one- or two-sided<br>*Only common tests should be described solely by name; describe more complex techniques in the Methods section.*
☐	☒	A description of all covariates tested
☒	☐	A description of any assumptions or corrections, such as tests of normality and adjustment for multiple comparisons
☐	☒	A full description of the statistical parameters including central tendency (e.g. means) or other basic estimates (e.g. regression coefficient) AND variation (e.g. standard deviation) or associated estimates of uncertainty (e.g. confidence intervals)
☐	☒	For null hypothesis testing, the test statistic (e.g. *F*, *t*, *r*) with confidence intervals, effect sizes, degrees of freedom and *P* value noted<br>*Give P values as exact values whenever suitable.*
☒	☐	For Bayesian analysis, information on the choice of priors and Markov chain Monte Carlo settings
☒	☐	For hierarchical and complex designs, identification of the appropriate level for tests and full reporting of outcomes
☒	☐	Estimates of effect sizes (e.g. Cohen's *d*, Pearson's *r*), indicating how they were calculated

Our web collection on statistics for biologists contains articles on many of the points above.

Software and code

Policy information about availability of computer code

Data collection	Merged Microarray-Acquired dataset (MMD) used in our study have been previously described.10 The datasets cover 11 major cancer types (bladder, breast, colon, liver, lung, kidney, melanoma, ovary, pancreas, prostate, and stomach), and comprise of 95 independent GEO studies (http://www.ncbi.nlm.nih.gov/geo) and a total of 8386 samples, either tumours or relevant normal tissues. Raw data for the 11 cancer types were independently pre-process using author-defined methods or RMA-normalization using the R library affy package.11 All the raw data were based on the GPL 570 microarray platform (Affymetrix Human Genome U133 Plus 2.0 Array) and were merged and adjusted by the Combat method using the R library inSilicoMergine package.12 Probes annotated with specific genes were collapsed to the maximum expression values, which were adopted for subsequent analyses.<br>Normalized RNA-Seq data based on Illumina HiSeq platform were extracted for the 11 cancer types and 14 cohorts from the Cancer Genome Atlas (TCGA; abbreviation: BLCA, BRCA, COAD, LIHC, LUAD, LUSC, KICH, KIRC, KIRP, OV, PAAD, PRAD, SKCM, STAD) using the bioinformatics tool Xena browser (https://xenabrowser.net/). Here, rectal adenocarcinoma was included in COAD, which was then used for comparison with colorectal tissues in MMDs. Raw RNA-Seq data were quantified using the root square error method (RSEM), and log2 transformed (RSEM+1). The associated clinical parameters, such as survival and molecular subtypes were obtained for comparative purpose. Finally, 6116 tumour (primary tumour) and normal (solid tissue normal) tissues were retrieved.<br>Raw RNA-Seq data in MET500 for the 11 epithelial carcinoma were downloaded from the database of Genotypes and Phenotypes, subsequently processed using RSEM,13,14 and then normalized using fragments per kilobase of transcript per million mapped reads (FPKM) and log2 transformed (FPKM+0.001). A total of 585 metastatic tumours from various cancer types and body locations (soft tissue, skin, prostate, pancreas, lymph node, lung, liver, colon, breast, brain, bone marrow, bladder, and adrenal gland) were analysed.
Data analysis	Data collection above and the pipeline used for the bioinformatics reanalysis is available at https://github.com/YangHongDai/Radiosensitivity_index

For manuscripts utilizing custom algorithms or software that are central to the research but not yet described in published literature, software must be made available to editors and reviewers. We strongly encourage code deposition in a community repository (e.g. GitHub). See the Nature Research guidelines for submitting code & software for further information.

Data

Policy information about availability of data

All manuscripts must include a data availability statement. This statement should provide the following information, where applicable:

- Accession codes, unique identifiers, or web links for publicly available datasets
- A list of figures that have associated raw data
- A description of any restrictions on data availability

1. TCGA: https://xenabrowser.net/
2. MMD: see the pipeline described in https://github.com/YangHongDai/Radiosensitivity_index

Field-specific reporting

Please select the one below that is the best fit for your research. If you are not sure, read the appropriate sections before making your selection.

☒ Life sciences ☐ Behavioural & social sciences ☐ Ecological, evolutionary & environmental sciences

For a reference copy of the document with all sections, see nature.com/documents/nr-reporting-summary-flat.pdf

Life sciences study design

All studies must disclose on these points even when the disclosure is negative.

Sample size	Merged Microarray-Acquired dataset (MMD) used in our study have been previously described.10 The datasets cover 11 major cancer types (bladder, breast, colon, liver, lung, kidney, melanoma, ovary, pancreas, prostate, and stomach), and comprise of 95 independent GEO studies (http://www.ncbi.nlm.nih.gov/geo) and a total of 8386 samples, either tumours or relevant normal tissues. Raw data for the 11 cancer types were independently pre-process using author-defined methods or RMA-normalization using the R library affy package.11 All the raw data were based on the GPL 570 microarray platform (Affymetrix Human Genome U133 Plus 2.0 Array) and were merged and adjusted by the Combat method using the R library inSilicoMergine package.12 Probes annotated with specific genes were collapsed to the maximum expression values, which were adopted for subsequent analyses.<br>Normalized RNA-Seq data based on Illumina HiSeq platform were extracted for the 11 cancer types and 14 cohorts from the Cancer Genome Atlas (TCGA; abbreviation: BLCA, BRCA, COAD, LIHC, LUAD, LUSC, KICH, KIRC, KIRP, OV, PAAD, PRAD, SKCM, STAD) using the bioinformatics tool Xena browser (https://xenabrowser.net/). Here, rectal adenocarcinoma was included in COAD, which was then used for comparison with colorectal tissues in MMDs. Raw RNA-Seq data were quantified using the root square error method (RSEM), and log2 transformed (RSEM+1). The associated clinical parameters, such as survival and molecular subtypes were obtained for comparative purpose. Finally, 6116 tumour (primary tumour) and normal (solid tissue normal) tissues were retrieved.<br>Raw RNA-Seq data in MET500 for the 11 epithelial carcinoma were downloaded from the database of Genotypes and Phenotypes, subsequently processed using RSEM,13,14 and then normalized using fragments per kilobase of transcript per million mapped reads (FPKM) and log2 transformed (FPKM+0.001). A total of 585 metastatic tumours from various cancer types and body locations (soft tissue, skin, prostate, pancreas, lymph node, lung, liver, colon, breast, brain, bone marrow, bladder, and adrenal gland) were analysed.
Data exclusions	Missing data about the genes involved or investigated were excluded
Replication	As RSI was derived from microarray datasets, its use in RNA-Seq platforms has never been elucidated. Prior to proceeding further, we first evaluated the quality of RSI application on TCGA and MMD using sigQC.15 sigQC is an R package for gene signature quality control, which encompasses a number of statistical metrics describing the ability of a gene signature to represent a dataset of interest, such as variability of signature genes and co-correlation of signature genes.
Randomization	Not relevant to our study as no randomization is needed
Blinding	Our study did not involve randomization and therefore blinding was not needed.

Reporting for specific materials, systems and methods

We require information from authors about some types of materials, experimental systems and methods used in many studies. Here, indicate whether each material, system or method listed is relevant to your study. If you are not sure if a list item applies to your research, read the appropriate section before selecting a response.

Materials & experimental systems

n/a	Involved in the study
☒	☐ Antibodies
☒	☐ Eukaryotic cell lines
☒	☐ Palaeontology and archaeology
☒	☐ Animals and other organisms
☐	☒ Human research participants
☒	☐ Clinical data
☒	☐ Dual use research of concern

Methods

n/a	Involved in the study
☒	☐ ChIP-seq
☒	☐ Flow cytometry
☒	☐ MRI-based neuroimaging

Human research participants

Policy information about studies involving human research participants

Population characteristics	The cancer data in our study were all retrieved from TCGA and GEO datasets. The relevant data about age, gender, sample types, and phenotypes were described in detail at individual websites. We used the open source data and extracted the RNA-Seq or microarray information for analyses in our study. Further detailed information could be referred to their websites.
Recruitment	See above
Ethics oversight	See above

Note that full information on the approval of the study protocol must also be provided in the manuscript.
